# Chromatin-focused genetic and chemical screens identify BRPF1 as a targetable vulnerability in Taxol-resistant triple-negative breast cancer

**DOI:** 10.1038/s12276-025-01466-5

**Published:** 2025-06-30

**Authors:** Ozlem Yedier-Bayram, Ahmet Cingöz, Ebru Yilmaz, Ali Cenk Aksu, Beril Esin, Nareg Degirmenci, Ayse Derya Cavga, Beyza Dedeoğlu, Buse Cevatemre, Hamzah Syed, Martin Philpott, Adam P. Cribbs, Udo Oppermann, Nathan A. Lack, Ceyda Acilan, Tamer T. Onder, Tugba Bagci-Onder

**Affiliations:** 1 Koç University Research Center for Translational Medicine, Istanbul, Turkey; 2 Biostatistics, Bioinformatics and Data Management Core, Koç University Research Center for Translational Medicine, Istanbul, Turkey; 3 Koç University School of Medicine, Istanbul, Turkey; 4 Botnar Research Centre, Nuffield Department of Orthopedics, Rheumatology and Musculoskeletal Sciences, National Institute of Health Research Oxford Biomedical Research Unit, University of Oxford, Oxford, UK; 5 Oxford Centre for Translational Myeloma Research, University of Oxford, Oxford, UK; 6 Vancouver Prostate Centre, University of British Columbia, Vancouver, British Columbia Canada; 7 Department of Medical Pharmacology, Koç University School of Medicine, Istanbul, Turkey

**Keywords:** Breast cancer, Cancer therapeutic resistance, Chemotherapy, Epigenetics

## Abstract

Triple-negative breast cancer (TNBC) is a particularly aggressive and frequently recurring form of breast cancer, where chemotherapy is the primary treatment approach. Unfortunately, the development of resistance to chemotherapy poses a considerable challenge, restricting the already limited therapeutic alternatives for recurrent cases. Here, we generated two Taxol-resistant TNBC cell lines with a dose-escalation method to mimic chemotherapy resistance in vitro. These cells exhibited reduced growth rates, altered morphology and evasion of apoptosis. Transcriptome analysis uncovered elevated *ABCB1* expression and multidrug-resistant phenotype in these resistant cells. To comprehensively investigate the key epigenetic regulators of Taxol resistance, we conducted chromatin-focused genetic and chemical screens and pinpointed Bromodomain and PHD Finger Containing 1 (BRPF1) as a novel regulator of Taxol resistance. Knockout of BRPF1, the reader protein in the MOZ–MORF histone acetyltransferase complex, but not the other complex members, sensitized resistant cells to Taxol. In addition, BRPF1 inhibitors, PFI-4 and OF-1, in combination with Taxol significantly reduced cell viability. Transcriptome analysis upon BRPF1 loss or inhibition revealed a negative impact on ribosome biogenesis-related gene sets, resulting in a global decrease in protein translation in Taxol-resistant cells. CUT&RUN-qPCR analysis demonstrated that BRPF1 directly binds to the *ABCB1* promoter, enhancing its expression toward inducing a multidrug-resistant phenotype. Conversely, knockout or inhibition of BRPF1 leads to decreased ABCB1 expression. Our findings uncover a comprehensive molecular framework, highlighting the pivotal role of epigenetic reader protein BRPF1 in Taxol resistance and providing potential avenues for therapeutic intervention in TNBC.

## Targeting BRPF1 offers hope against Taxol resistance

Triple-negative breast cancer (TNBC) is a challenging type of breast cancer that lacks targeted treatment options. Researchers aimed to understand why some TNBC cells become resistant to the chemotherapy drug Taxol and focused on the role of a protein called BRPF1 in this resistance. They created Taxol-resistant TNBC cells in the laboratory and found that these cells had high levels of a protein called ABCB1, which helps to pump drugs out of cells. They used advanced techniques to identify key proteins involved in this resistance and discovered that BRPF1, part of a complex that modifies DNA structure, plays a crucial role. By using genetic tools and chemical inhibitors to reduce BRPF1 activity, they found that the resistant cells became more sensitive to Taxol. This suggests that targeting BRPF1 could be a new way to treat resistant TNBC.

This summary was initially drafted using artificial intelligence, then revised and fact-checked by the author.

## Introduction

Triple-negative breast cancer (TNBC) is a very aggressive and recurrent type of breast cancer that predominantly affects younger women^1^. TNBC tumors are characterized by the absence of estrogen receptor (ER), progesterone receptor (PR) and human epidermal growth factor receptor-2 (HER2) overexpression. The lack of receptor expression limits targeted therapy options, leaving taxane- and anthracycline-based chemotherapy as the mainstay treatments^2^.

Taxol (paclitaxel), a member of the taxane class, exerts its therapeutic effect by inducing mitotic catastrophe. It binds to and stabilizes microtubules, preventing their disassembly during metaphase, resulting in mitotic arrest at the G_2_/M checkpoint and eventual cell death^3,4^. Although patients with TNBC initially respond to taxane-based chemotherapy, they are more prone to developing resistance and recurrence than hormone receptor-positive counterparts^2,5^. Taxol resistance is often linked to increased expression of ABC transporters, particularly ABCB1 (P-glycoprotein, MDR1), which actively pumps the drugs out of the cell. The broad substrate specificity of the ABC transporters limits the use of alternative chemotherapeutics once they are overexpressed, leading to a multidrug resistance (MDR) phenotype^6^. Efforts to overcome MDR through the combined use of ABC transporter inhibitors and chemotherapeutics have faced challenges^7^. First-generation ABCB1 inhibitors, such as verapamil, resulted in toxic side effects without additional benefits^8^. Although second-generation inhibitors aimed to enhance specificity and reduce side effects, they inadvertently increased systemic exposure, exacerbating toxicity. Third-generation ABCB1 inhibitors, despite effectively reversing ABCB1-mediated MDR, did not improve overall survival rates across various cancer types^9–11^. Moreover, the possibility of compensation through the co-expression of different ABC transporter family members complicates targeted inhibition strategies. Therefore, understanding the cancer specific upstream regulators of ABC transporters is necessary to circumvent the toxicity linked with direct inhibition of ABCB1.

Advances in single-cell sequencing technologies have revealed nongenetic mechanisms and preexisting epigenetically poised cells that contribute to intratumor heterogeneity and clonal selection during treatment^12–15^. For instance, heterogeneity in histone modifications, such as H3K27me3, has been linked to the emergence of Taxol-resistant subpopulations^16^. EZH2 inhibitors that decrease histone methylation sensitized resistant cells to paclitaxel and reduced metastasis^16–18^. Conversely, it has been shown that H3K27me3 prevents cells from escaping chemotherapy, acting as a lock in drug-tolerant persister cell-specific genes in TNBC cells^19^. Inhibitors of H3K27me3 demethylases, combined with 5-Fluorouracil, reduced the number of drug-tolerant cells^19^. Preexistence of drug-tolerant tumor cells in populations with increased levels of KDM5A and hypersensitivity to HDAC inhibitors has also been shown^20^. Chromatin remodeling factors regulating the accessibility of stemness genes in TNBC contribute to invasive tumors^21^. Alternatively, chromatin regulators such as BAF and COMPASS complexes and KDM4B have been found to increase anthracycline sensitivity by enhancing chromatin accessibility^22^. Collectively, these findings highlight the notable impact of epigenetic regulators on the development of chemoresistance, while their specific roles might be tissue, cancer and time dependent.

The link between epigenetic regulation of Taxol resistance and the MDR phenotype remains poorly defined. Earlier studies showed that loss of repressive marks on the *ABCB1* promoter, such as DNA methylation, leads to increased expression of ABCB1 in different cancer types^23–26^. A correlation between increased KDM5A levels and ABCB1 expression was demonstrated in lung adenocarcinoma^27^. Meanwhile, a recent study proposed that higher-order three-dimensional genome topology is crucial for *ABCB1* activation^28^. While modulation of DNA methylation or histone acetylation had no clear impact on ABCB1 expression, dissociation of the *ABCB1* locus from the nuclear lamina led to its activation in Taxol-resistant cells. These data underscore the absence of a clear consensus on how ABCB1 is epigenetically regulated, emphasizing the need for further investigation.

In this study, we examined the epigenetic modifiers regulating Taxol resistance in TNBC cells by first generating Taxol-resistant cells in vitro. Characterization of resistant cells revealed that *ABCB1* was highly upregulated. Using our chromatin-focused CRISPR–Cas9 library, EPIKOL^29^, and an epigenetic probe library concurrently, we identified epigenetic vulnerabilities in Taxol-resistant cells. Bromodomain and PHD Finger Containing 1 (BRPF1) protein, a member of a histone acetyltransferase (HAT) complex, emerged as a regulator of Taxol resistance and ABCB1 expression. Transcriptome analysis also demonstrated that BRPF1 contributes to elevated ABCB1 protein levels and regulates ribosome biogenesis, impacting protein translation. Therefore, the combination of BRPF1 inhibitors with chemotherapy for ABCB1-high tumors presents a promising therapeutic opportunity.

## Methods

### Cell culture

SUM159PT TNBC cell line and HEK293T cells were kind gifts from Robert Weinberg (MIT, Boston, USA). HEK293T cells were cultured in Dulbecco’s modified Eagle medium (Gibco) supplemented with 10% fetal bovine serum (Gibco) and 1% penicillin–streptomycin (Gibco). SUM159PT cells were cultured in Ham’s F12 nutrient mix (Gibco) supplemented with 5% FBS, 5 µg/ml insulin (Sigma-Aldrich), 1 µg/ml hydrocortisone (Sigma-Aldrich) and 10 mM HEPES (Thermo Fisher). All cell lines were tested regularly for mycoplasma infection.

### Cell viability assay and determination of IC_50_ values

Cells were seeded into 96-well black plates at 2,000 cells per well for SUM159PT cells and its resistant derivatives. The next day, the cells were treated with the corresponding chemicals. For half-maximal inhibitory concentration (IC_50_) determination, Taxol (paclitaxel, Sigma) was serially diluted 3.16-fold (starting from 10 µM to 0.1 nM). After 72 h of treatment with the indicated chemical, media were discarded, and a luminescence-based cell viability assay (CellTiter-Glo, Promega) was performed according to the manufacturer’s recommendations. The results were analyzed using GraphPad Prism 8.

### Generation of drug-resistant TNBC cell lines

Cells were seeded at 2 × 10^5^ cells per well in 6-well plates. The next day, they were treated with either 10% inhibitory concentration (IC_10_)(0.4 nM) or IC_50_ (1.5 nM) values of Taxol for 72 h as starting concentrations. When cells became confluent under drug treatment, concentrations were doubled. If cells did not look healthy or were too sparse, they were placed into drug-free medium until they formed colonies. Upon reaching confluence, the last concentration of drug was applied again, and the procedure was repeated. Alongside this, dimethyl sulfoxide (DMSO)-treated parental cell lines were passaged as controls. Drug treatment was continued until a significant difference between IC_50_ values of parental and resistant cell lines was obtained. Resistant cells derived from SUM159PT cells were named according to the initial and final doses of Taxol applied. Cells were maintained at the final dose of Taxol (160 nM for T1-160 and 450 nM for T2-450) while culturing.

### Immunofluorescence

Cells were fixed with ice-cold 100% methanol for 10 min at −20 °C. After washing with phosphate-buffered saline twice, cells were permeabilized with 0.01% Triton X-100 at room temperature for 5 min. Cells were blocked with SuperBlock (ScyTek Laboratories) for 15 min at room temperature and incubated with the primary antibody anti-α-tubulin (DM1A) (Sigma T9026) 1:10,000 diluted in SuperBlock for 1 h at room temperature. After washing, cells were incubated with Alexa Fluor 488 anti-mouse secondary antibody (Invitrogen) for 1 h at room temperature in the dark, and coverslips were mounted with DAPI (mounting medium with DAPI, Abcam, #ab104139). Images were taken using Zeiss Axio Imager M1 at 40× magnification.

### Epigenetic probe library screen

The chemical probe library targeting a wide range of epigenetic modifiers consists of 117 drugs (Supplementary Table 3). The library was constructed as described^30^. Cells were seeded at 750 cells per well in 384-well plates. The next day, the cells were treated with epigenetic probes alone or in combination with Taxol at a final concentration of 2.5 nM for parental, 300 nM for T1-160 and 500 nM for T2-450. The Taxol doses were selected to correspond to 40–50% inhibitory concentration (IC_40_–IC_50_)in 384-well plate format. Epigenetic probes were added at 1:1,000 dilution in triplicate at the final concentrations indicated in Supplementary Table 3. 72 hours later, the cell viability was measured by using CellTiter-Glo, and the results were normalized to untreated controls. The mean cell viability and standard deviations (s.d.) of DMSO controls (in combination with Taxol) were calculated as 79.67% ± 0.7% for parental, 54.24% ± 1.43% for T1-160 and 62.20% ± 1.02% for T2-450 screens. An epigenetic probe was considered a ‘hit’ if it decreased cell viability by more than 3 s.d. of DMSO in combination with Taxol (77.57% for parental, 49.95% for T1-160 and 59.15% for T2-450). The hits were excluded if their inactive compound controls also affected cell viability.

### Chromatin-focused knockout screen

Epigenetic Knockout Library (EPIKOL), which targets 779 genes, including several negative and positive controls, was used, and viruses were generated as described^29^. After Cas9-expressing T1-160 cells were generated, pooled lentiviral EPIKOL was transduced as 1,000× representation with a multiplicity of infection (MOI) of 0.4. Transduced cells were selected with 5 µg/ml puromycin in the presence of 20 µM verapamil for 4 days, and 8 × 10^6^ cells were collected as the initial time point. Cells were divided into DMSO-treated or 160 nM Taxol-treated groups, cultured until they reached 16 population doublings, and final cell pellets were collected at 8 × 10^6^. Genomic DNA was isolated using the Nucleospin Tissue kit (Macherey-Nagel) according to the manufacturer’s instructions. Preparation of next-generation sequencing libraries and analysis of the results were performed as described^29^. A *P* < 0.05 cutoff was applied to gene-level analysis to identify significantly depleted genes.

### Dual-color competition assays

For validation of EPIKOL screen candidate hits, dual-color competition assays were performed as described using T1-160-Cas9 cells^29^. A list of single guide RNAs (sgRNAs) can be found in Supplementary Table 4. During the analysis, one group was treated with 160 nM Taxol, whereas the other was treated with DMSO as a vehicle. Cells were incubated for 24 days, with images taken on days 0, 4, 8, 12, 16 and 24. The number of mCherry^+^ and eGFP^+^ cells was counted from images using Gen5 software (BioTek), and each measurement was normalized to day 0 to determine the percentage of GFP-positive cells.

### siRNA experiments

T1-160 cells were seeded at 300.000 cells per well in 6-well plates. The next day, Lipofectamine 3000 (Invitrogen) transfection was performed. In brief, one mixture containing 125 µl Opti-MEM with 7.5 µl Lipofectamine 3000 reagent and another mixture containing 125 µl Opti-MEM with 100 pmol of either BRPF1 small interfering RNA (siRNA; Thermo Fisher Scientific, cat no: 4392421, siRNA ID: s15422) or nontargeting siRNA (siNT) were prepared and vortexed well. siRNA mixture was added onto the Lipofectamine 3000 mixture and incubated for 15 min. The final mixture was added to the cells dropwise, and 8 h later, fresh media were added. The next day, cells were trypsinized and seeded for cell viability or colony formation assays.

### Functional rescue experiment

For the overexpression of BRPF1 on cells carrying BRPF1 sgRNA, BRPF1 was cloned into a lentiviral vector, and the protospacer adjacent motif (PAM) sequence adjacent to the sgRNA binding site was mutated. For this, GFP-BRPF1 plasmid (Addgene #65382) was used as a template and amplified by using forward primers containing SalI and reverse primers containing XbaI cut sites. The resulting PCR product was cut and ligated to pENTR1A (Addgene #17398) entry vector. Then, Gateway cloning was performed to clone BRPF1 into pLEX_305-C-dTAG (Addgene #91798). The PAM sequence adjacent to the BRPF1 sgRNA #1 was changed from NGG to NAG to ensure that the overexpression construct will not be targeted by CRISPR–Cas9. Later, T1-160 cells were infected with BRPF1 sgRNA and the overexpression construct at the same time. On post-transduction day 5, cells were seeded for clonogenic assay.

### SUnSET assay

To measure the global translation rate, SUnSET assay, which nonradioactively measures puromycin-labeled peptide amounts in a given period^31^, was utilized. For this, T1-160 cells were infected with NT1 or BRPF1-targeting sgRNAs in the LentiGuide backbone with hygromycin selection. On day 10 after transduction, T1-160-NT1 cells were treated with cycloheximide (5 µM) as a positive control for 48 h. On day 12, T1-160 cells carrying NT1 or BRPF1-targeting sgRNAs were treated with puromycin (50 µg/ml) in the presence of verapamil (20 µM) for 30 min to allow the incorporation of puromycin into newly synthesized polypeptides. After cell lysis and protein isolation, 20 µg of total protein per sample was subjected to western blot analysis using an anti-puromycin primary antibody to detect puromycin-labeled nascent polypeptides, thereby reflecting the global translation rate of the cells.

### CUT&RUN-qPCR

For cleavage under targets and release using nuclease (CUT&RUN) experiments, we overexpressed BRPF1 with double-HA tag, whose cloning is explained above (see ‘Functional rescue experiment’ section). T1-160 cells were infected with lentiviral construct containing BRPF1-HA to and selected with puromycin. CUT&RUN was performed using CST CUT&RUN assay kit (#86652) according to the manufacturer’s recommendations. To detect histone acetylation changes, T1-160 cells infected with BRPF1 sgRNA were used on PT8. Anti-HA.11 antibody (16B12, BioLegend, 1:25), acetyl-histone H3 (Lys27) antibody (#4353, CST, 1:25), acetyl-histone H3 (Lys14) antibody (#7627, CST, 1:50), and acetyl-histone H3 (Lys9) antibody (#9649, CST, 1:50) were used. Primers used in CUT&RUN-qPCR are listed in Supplementary Table 5. Results were calculated using the % input method and spike-in DNA normalization.

### Statistical analysis

Analysis of EPIKOL data was performed by using the robust rank aggregation method in MAGeCK. Comparisons between two groups were performed using a two-tailed Student’s *t*-test for normally distributed data; otherwise, the Mann–Whitney *U* test was used. For comparisons involving more than two groups, one-way or two-way analysis of variance (ANOVA) with Tukey’s post hoc test was applied for parametric data using Prism 8 (GraphPad Software). Significance levels were set as *P < 0.05, **P < 0.01, ***P < 0.001.

## Results

### Taxol-resistant TNBC cells demonstrate the characteristics of chemotherapy resistance

**Fig. 1:**
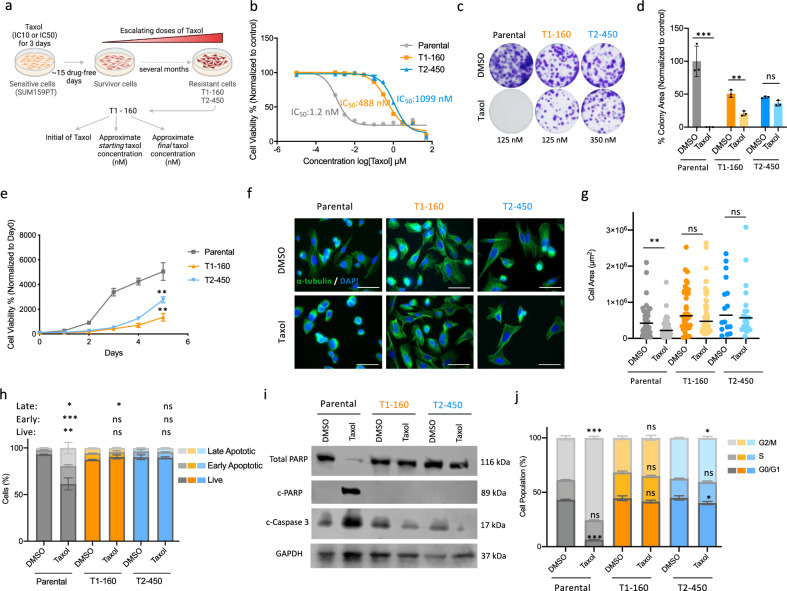
Establishment and characterization of Taxol-resistant TNBC cells. **a** A schematic representation of the protocol used for generating Taxol-resistant SUM159PT cells. The cells were exposed to increasing concentrations of Taxol (IC_10_ or IC_50_) for 72 h, with subsequent doubling of the drug amount upon confluence. This cycle was repeated until the IC_50_ values of the cells significantly differed from the initial cell population. Created with BioRender.com. **b** IC_50_ values for T1-160 (generated as a resistant cell to the folds of IC_10_ value of Taxol) and T2-450 (resistant to the folds of IC_50_). **c** Colony formation assay in the presence of indicated amounts of Taxol. **d** Quantification of colony areas in **c**. **e** A comparison of growth rates between parental and Taxol-resistant SUM159PT cells. **f** Immunofluorescence staining for α-tubulin (green) and DAPI (blue) after 4 h of exposure to DMSO or Taxol (parental: 160 nM, T1-160: 160 nM, T2-450: 450 nM). Scale bar: 50 µm. **g** Quantification of cell area in **f**. **h** AnnexinV/Dead cell assay after 24 h of DMSO or Taxol treatment (parental: 160 nM, T1-160: 160 nM, T2-450: 450 nM). **i** Western blot analysis of the cells shown in **h** for total PARP, cleaved-PARP (c-PARP), cleaved-caspase3 (c-Caspase 3) and GAPDH as a loading control. **j** Cell cycle assay after 8 h of DMSO or Taxol treatment (parental: 160 nM, T1-160: 160 nM, T2-450: 450 nM). For statistical analysis, each Taxol group was compared with same cell’s DMSO group. *P* values determined by two-tailed Student’s *t*-test in comparison with control group; *P < 0.05, **P < 0.01, ***P < 0.001.

To mimic chemotherapy resistance in vitro, we generated Taxol (paclitaxel)-resistant derivatives from SUM159PT TNBC cells. First, we measured the viability of SUM159PT cells in the presence of increasing doses of Taxol and determined IC_10_ and IC_50_ values. Then, to generate Taxol-resistant cells, SUM159PT cells were treated with IC_10_ and IC_50_ doses of Taxol for 3 days (Fig. 1a). Depending on the viability of the cells, they were either kept in drug-free fresh media or treated with the same dose of Taxol until they were confluent. Once the cells lost sensitivity to the drug, the amount of Taxol was doubled. Two different Taxol-resistant SUM159PT cell lines were generated by this method and named according to the starting and final doses of Taxol (Fig. 1a). T1-160 and T2-450 cells were both highly resistant to Taxol as indicated by the increase in their IC_50_ values compared with the parental cells (Fig. 1b). Long-term colony formation assay in the presence of Taxol clearly demonstrated that the T1-160 and T2-450 cells can survive under high doses of Taxol treatment whereas the parental cells cannot (Fig. 1c, d). Resistant cells grew significantly slower than the parental cells (Fig. 1e). Immunofluorescence staining of α-tubulin on parental and resistant cells demonstrated that resistant cells were larger in size compared with parental cells. While the morphology and microtubule organization of the parental cells markedly changed upon Taxol treatment, resistant lines were not affected, as indicated by bright-field microscopy and α-tubulin immunofluorescence staining (Fig. 1f, g and Supplementary Fig. 1a). Competition assay of parental and resistant cells similarly showed that only resistant cells were able to grow in the presence of Taxol (Supplementary Fig. 1b). Annexin V/Dead cell assay demonstrated that the number of apoptotic cells was significantly higher in parental cells with Taxol treatment, while there was minimal change in resistant cells (Fig. 1h). Furthermore, cleaved-PARP and increased cleaved-caspase-3 were observed only in Taxol-treated parental cells but not in resistant cells (Fig. 1i). As the mechanism of action of Taxol is to stabilize microtubules, leading to cell cycle arrest at the G_2_/M phase, we next assessed the cell cycle distribution patterns of Taxol-treated cells. In line with previous results, Taxol-treated parental cells significantly accumulated in the G_2_/M phase, and no major change was observed in resistant cells (Fig. 1j). T1-160 and T2-450 cells were also cross-resistant to doxorubicin and vincristine. Whereas vincristine has a similar mode of action to Taxol, doxorubicin has a completely different mechanism in which it inhibits TOP2B, leading to DNA damage (Supplementary Fig. 1c).

Altogether, these data showed that we successfully generated Taxol-resistant SUM159PT cells by the dose-escalation method, and these cells demonstrate the characteristics of chemotherapy-resistant cells.

### Transcriptomic changes of Taxol-resistant TNBC cells highlight ABCB1-mediated resistance

**Fig. 2:**
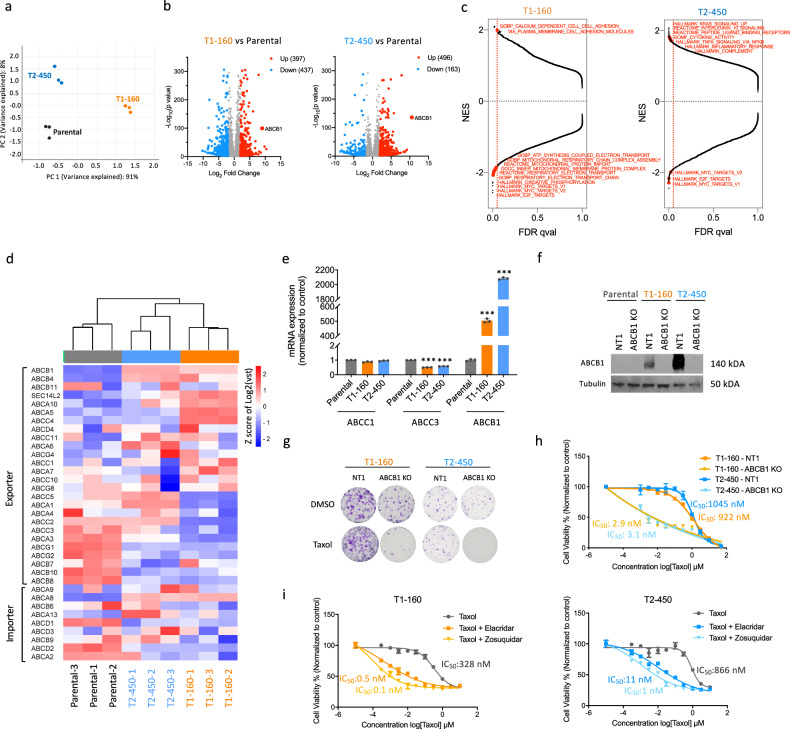
RNA sequencing results of SUM159PT Taxol-resistant cells and characterization of ABCB1-upregulated phenotype. **a** Principal component analysis (PCA) plot showing the distribution of parental and Taxol-resistant SUM159PT cells after RNA sequencing. **b** Volcano plots displaying differentially expressed genes in T1-160 (left) and T2-450 (right) cells compared with parental cells, with a log_2_-fold change (LFC) >2 and *P* < 0.001 cutoff. **c** Gene set enrichment analysis (GSEA) on preranked gene lists based on LFC values for T1-160 (left) and T2-450 (right) cells. **d** A heatmap illustrating the gene expression pattern of multidrug transporter family members in parental and resistant cells. **e** qPCR validations of mRNA expression for various multidrug transporter genes. **f** Western blot analysis of ABCB1 protein expression in parental and resistant cells with and without ABCB1-targeting sgRNA. **g** Clonogenic assay conducted on ABCB1-KO resistant cells in the presence of Taxol. **h** Cell viability assay performed on ABCB1-KO resistant cells in the presence of Taxol. **i** Cell viabilities of resistant cells upon elacridar and zosuquidar treatment with increasing doses of Taxol. *P* values determined by two-tailed Student’s *t*-test in comparison with control group; ***P < 0.001.

To elucidate the underlying mechanisms of resistance, transcriptomes of Taxol-resistant TNBC cell lines were analyzed by RNA sequencing. Principal component analysis demonstrated that triplicates of each cell line formed distinct clusters, separate from other parental or resistant cell lines (Fig. 2a). Comparison of T1-160 cells with the parental cells revealed 397 upregulated and 437 downregulated genes with a log_2_-fold change>2 and a significance level of *P* < 0.001 (Fig. 2b). In T2-450 cells, 496 upregulated and 163 downregulated genes were identified (Fig. 2b).

Gene set enrichment analysis in T1-160 cells revealed a predominance of negatively enriched gene sets (false discovery rate *q* value <0.05), particularly those associated with oxidative phosphorylation and the electron transport chain, indicating a potential shift toward increased glycolysis compared with parental cells. Positively enriched gene sets in T1-160 cells were linked to cell adhesion. By contrast, T2-450 cells exhibited more positively enriched gene sets, with a focus on inflammatory response and the complement system (Fig. 2c).

Analyzing the most significantly upregulated and downregulated genes (Fig. 2b), we identified ABCB1 as the top upregulated gene in both resistant cell lines. As a member of the ABC transporter family, ABCB1 is widely recognized for its strong association with drug resistance^32^. This finding aligns with previous cross-resistance results observed in the resistant cells (Supplementary Fig. 1c), reinforcing the presence of an MDR phenotype. A comprehensive overview of the ABC transporter landscape showed that, in both resistant cell lines, the majority of exporters exhibited upregulation, while several were downregulated (Fig. 2d). Concurrently, a considerable portion of importers showed downregulation, suggesting an adaptive mechanism aimed at preventing the entry of chemotherapeutic agents into the cells. Notably, ABCB1 demonstrated significant overexpression at the RNA and protein levels (Figs. 2e,f). Given that the copy number variations (CNVs) of ABCB1 are commonly linked to its elevated expression^33^, we assessed the ABCB1 copy number in the genomic DNAs of resistant cells and identified an increase in ABCB1 copy number in both cell lines (Supplementary Fig. 2a). ABCB1 knockout (KO) in resistant cell lines completely eradicated ABCB1 expression (Fig. 2f). Clonogenic assays conducted with ABCB1-KO cells clearly demonstrated their inability to form colonies under Taxol pressure (Fig. 2g). As expected, IC_50_ values for Taxol were significantly lower in ABCB1-KO cells than controls (Fig. 2h). Moreover, we treated the resistant cells with three different ABCB1 inhibitors. Treatment with verapamil, a first-generation ABC transporter inhibitor^6^ and calcium channel blocker, significantly lowered IC_50_ values for Taxol (Supplementary Fig. 2b). Fluorescence imaging of calcein-AM further illustrated that the uptake of calcein was restricted in Taxol-resistant cells but augmented in the presence of verapamil, indicating the functional role of ABCB1 in these resistant cells (Supplementary Fig. 2c). Elacridar, a second-generation ABC inhibitor that selectively binds to several ABC proteins, and zosuquidar, a third-generation ABC inhibitor with high affinity for ABCB1^34^, both reversed resistance, with the most pronounced effect observed with zosuquidar (Fig. 2i). This set of data underscores the profound impact of ABCB1 on the resistance of these cells.

### Chromatin-focused screens reveal epigenetic vulnerabilities of resistant cells

**Fig. 3:**
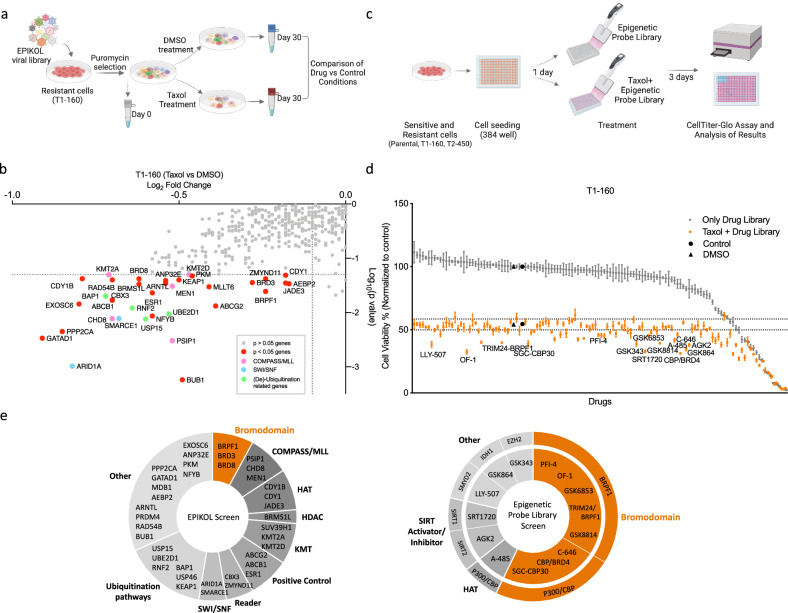
Chromatin-focused genetic and chemical probe library screens reveal epigenetic vulnerabilities of Taxol-resistant TNBC cells. **a** A schematic representation of EPIKOL screen on T1-160 resistant cells. Cells were infected with 1,000× representation of library with an MOI of 0.3 and selected with puromycin. After completion of Cas9 activity approximately 9 days after transduction, cells were divided into two groups as DMSO-treated and Taxol-treated (160 nM) groups. The final time-point samples were compared to identify epigenetic modifiers that sensitize Taxol-resistant cells. Created with BioRender.com. **b** Results of the EPIKOL screen performed on T1-160 cells, presented as LFC of genes in Taxol-treated samples compared with the DMSO-treated group. Genes that have *P* < 0.05 were colored and labeled. **c** A schematic representation of the epigenetic chemical probe library screen. Cells were seeded to 384-well plates at 750 cells per well and the next day treated with Taxol (parental: 1.5 nM, T1-160: 300 nM, T2-450: 500 nM) and 1× concentration of epigenetic probe library. After 3 days, cell viability was measured. Created with BioRender.com. **d** Results of the epigenetic probe library screen performed on T1-160 cells. The gray dots represent the effect of epigenetic probes alone, while the orange dots show changes in cell viability when Taxol was combined with a specific epigenetic probe. Epigenetic probes that significantly reversed drug resistance are labeled in the graph. If the negative control of a given probe also showed a significant depletion in cell viability, neither probe was labeled on the graph. **e** Classification of hits identified in genetic (left) and chemical (right) screens. For the chemical screen, the target genes of chemical probes are indicated in the outer circle.

To uncover epigenetic regulators of Taxol resistance, we utilized two complementary approaches: epigenome-wide CRISPR–Cas9 and epigenetic probe library screens. For the CRISPR–Cas9 library screen, we took advantage of our previously published chromatin-focused sgRNA library (EPIKOL)^29^. After infection, culturing and sequencing, the Taxol-treated T1-160 cells were compared with the DMSO-treated group to identify epigenetic modifiers whose loss revert resistance (Fig. 3a). ABCB1 and ABCG2, positive controls present in our library, were highly depleted in the presence of Taxol, validating the reliability of our screen. Notably, members of the COMPASS–MLL complex, SWI–SNF complex and deubiquitination-related genes were among the most significantly depleted genes (Fig. 3b).

As an alternative approach, we utilized an epigenetic probe library consisting of 117 chemical probes targeting a broad range of epigenetic factors (Supplementary Fig. 3a). Parental and resistant cells were treated with Taxol alone or in combination with epigenetic probes. After 72 h of treatment, cell viability was assessed (Fig. 3c). The majority of the epigenetic probes exhibited minimal impact on T1-160 cell viability individually (Fig. 3d). Various bromodomain inhibitors, SIRT activator and inhibitors, mIDH1 inhibitor and EZH2 inhibitor significantly decreased cell viability when applied together with Taxol (Fig. 3d).

Furthermore, we performed the probe library screen on SUM159PT parental and T2-450 Taxol-resistant cells (Supplementary Fig. 3b, c). Similar to the T1-160 cells, numerous bromodomain inhibitors, particularly those targeting BRPF1, and SIRT activator and inhibitors affected cell viability when combined with Taxol. Interestingly, members of the lysine methyltransferase (KMT) family exhibited sensitizing effects specifically in T2-450 cells (Supplementary Fig. 3d). The majority of the epigenetic probes that reduced cell viability in combination with Taxol were shared between T1-160 and T2-450 cells (Supplementary Fig. 3e). Notably, inhibitors such as PFI-4, OF-1, GSK6853 and TRIM24–BRPF1 demonstrated a substantial impact on cell viability when combined with Taxol in resistant cells. By contrast, these BRPF1 inhibitors did not have a significant effect on parental cells.

To prioritize the hits from genetic and chemical screens, we classified the genes and inhibitors according to their complexes or epigenetic modifier classes (Fig. 3e). EPIKOL screen hit genes (*P* < 0.05) were classified into nine main categories: bromodomain-containing proteins, COMPASS–MLL complex, HAT, histone deacetylase (HDAC), KMT, readers, SWI–SNF complex, ubiquitination pathways and others. Epigenetic probe library screen hit targets include BRPF1 and p300–CBP of bromodomain family proteins, SIRT1, SIRT2, SMYD2, IDH1 and EZH2. Notably, when both of the screen results are considered together, BRPF1, a member of the bromodomain family, emerged as the sole target identified as a hit in both screens.

### EPIKOL screen hits are validated through functional assays

**Fig. 4:**
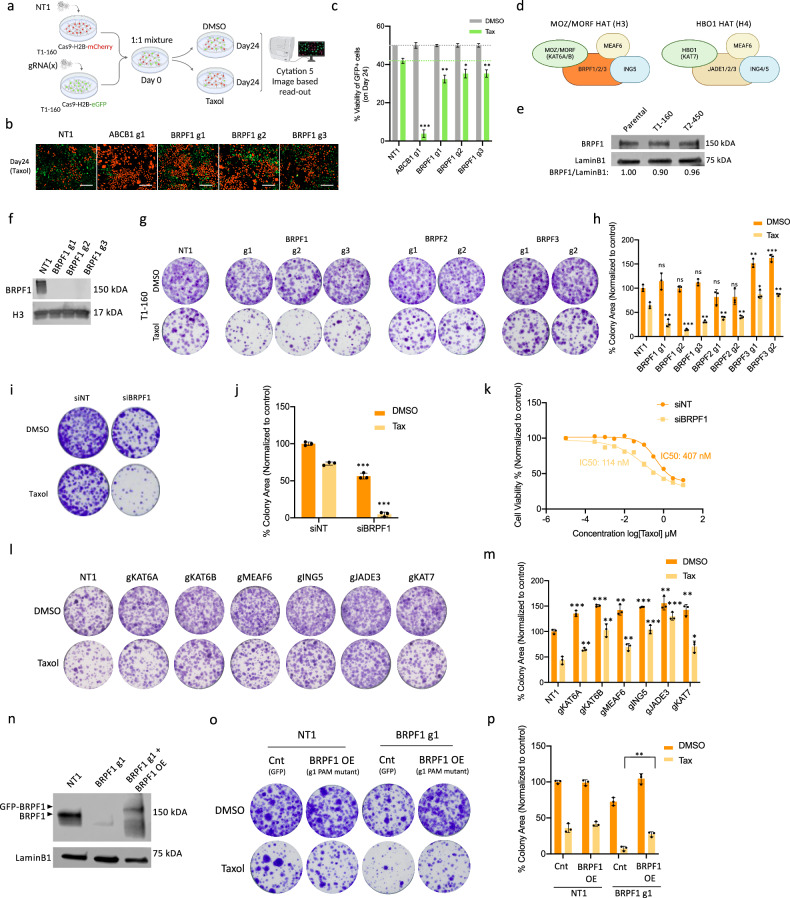
Genetic perturbation of BRPF1 sensitizes Taxol-resistant T1-160 cells. **a** A schematic representation of dual-color competition assay. mCherry-H2B- or eGFP-H2B-labeled T1-160 cells were infected with NT1 or sgRNA of interest. Cells were mixed in a 1:1 ratio, treated with Taxol (160 nM) and imaged after attachment and every 4 days until day 24. The ratio of eGFP-H2B-T1-160 cells carrying the sgRNA of interest was determined to identify the genes whose loss revert resistance. Created with BioRender.com. **b** Representative images of dual-color competition assay for KOs of indicated genes. Scale bar, 200 µm. **c** Statistical analysis of eGFP^+^ cells upon ABCB1 and BRPF1 KO after dual-color competition assay. **d** Complexes that are formed by BRPF and JADE proteins. Created with BioRender.com. **e** Western blot analysis of BRPF1 protein levels in parental and resistant cells. **f** Western blot analysis of BRPF1 expression upon BRPF1 KO in T1-160 cells. **g** Clonogenic assay showing the effect of BRPF1, BRPF2 and BRPF3 KOs in the presence of Taxol in T1-160 cells. **h** Quantification of the colonies in **g**. **i** The effect of BRPF1 siRNA on colony-forming abilities of T1-160 cells in the presence of Taxol. **j** Quantification of colonies in **i**. **k** Cell viability measurement performed with siNT and siBRPF1 samples in the presence of Taxol in T1-160 cells. **l** The effect of MOZ–MORF complex members’ KOs on colony-forming abilities of T1-160 cells in the presence of Taxol. **m** Quantification of colonies in **l**. **n** BRPF1 protein levels upon KO and overexpression of PAM mutant version of GFP fused BRPF1 in T1-160 cells **o**, Rescue experiment with overexpression of BRPF1 PAM mutant version on BRPF1-KO cells in the presence of Taxol. **p** Quantification of colonies in **o**. *P* values determined by two-tailed Student’s *t*-test. For each condition (DMSO or Taxol), statistical comparisons were made relative to their respective control groups. ; *P < 0.05, **P < 0.01, ***P < 0.001.

Genes that have high log-fold changes and that are not previously associated with chemotherapy resistance were selected for further validation (Fig. 3b and Supplementary Fig. 4a). BRPF1 was included in this group as it was the only gene that was identified in both screens. To validate EPIKOL screen hits, we performed a dual-color cell growth competition assay (Fig. 4a). For this, mCherry-labeled T1-160-Cas9 stable cells were infected with NT1 and mixed with eGFP-labeled T1-160 Cas9 stable cells carrying the sgRNA of interest in a 1:1 ratio. For 24 days, cells carrying sgRNAs against the selected hits were outcompeted by the cells carrying nontargeting sgRNA (NT1). ABCB1 KO served as a positive control, exhibiting the most pronounced effect in the presence of Taxol (Fig. 4b, c). In this assay, KOs of GATAD1, PPP2CA, CHD8, BRD8, SMARCE1, KMT2A and MEN1 resulted in significant decreases in cell viability (Supplementary Fig. 4b). Despite BRPF1 not being the top scorer in the EPIKOL screen, it consistently exerted a small yet significant effect on cell viability in combination with Taxol. This phenotype was validated with three independent sgRNAs targeting BRPF1 (Fig. 4b, c). Given that BRPF1 was the only hit demonstrating a phenotypic effect through both genetic (KO) and pharmacological (inhibition with small compounds) approaches, we chose to further focus on BRPF1 as a potential regulator of chemoresistance.

### KO of BRPF1 sensitizes Taxol-resistant cells

BRPF1 (also known as peregrin and BR140) is a member of the HAT complex containing monocytic leukemia zinc finger protein (MOZ, KAT6A) or monocytic leukemia zinc finger protein-related factor (MORF, KAT6B) as catalytic subunits (Fig. 4d)^35^. BRPF1 is a chromatin reader that contains a plant homeodomain (PHD)–zinc-knuckle–PHD (PZP) module at the N-terminus, followed by a bromodomain recognizing acetyl-lysines, and a C-terminal proline-tryptophan–tryptophan–proline (PWWP) domain. BRPF1 acts as a scaffold by binding to MOZ–MORF through its N-terminus motifs and ING5 and MEAF6 through the downstream motif^36,37^. Initially, we assessed mRNA and protein levels of BRPF1 and observed that resistant cells show only a slight decrease or no change in BRPF1 levels (Fig. 4e and Supplementary Fig. 5a). Despite modest decreases in mRNA levels with BRPF1-targeting sgRNA expression, BRPF1 protein was completely lost in KO samples (Fig. 4f and Supplementary Fig. 5b). Notably, BRPF1 has two structurally close proteins, BRPF2 (BRD1) and BRPF3, which are known to be part of the MOZ–MORF complex^38^. Although BRPF2 and BRPF3 sgRNAs were not depleted during the EPIKOL screen, to exclude the possibility of their involvement in Taxol resistance, we analyzed the effects of individual BRPF2 and BRPF3 KOs on Taxol resistance (Supplementary Fig. 5c). As indicated by the clonogenic assay, the most significant effect in the presence of Taxol was observed in BRPF1-KO samples, with BRPF2 and BRPF3 KOs having minor or no effects (Fig. 4g, h). In the second resistant line, T2-450, loss of BRPF2 and BRPF3 did not show any effects, whereas BRPF1 was essential for cell survival (Supplementary Fig. 5d, e). These findings suggest that the sensitization effect to Taxol is attributed primarily to the KO of BRPF1.

As an independent and rapid loss-of-function approach, we utilized siRNA targeting BRPF1, which resulted in a substantial reduction in BRPF1 mRNA levels within a short timeframe (24–48 h) (Supplementary Fig. 5f). This rapid downregulation mirrored the sensitization phenotype observed in KO samples in the presence of Taxol (Fig. 4i, j). The acute suppression of BRPF1 enabled us to assess sensitization in siBRPF1 samples through an ATP-based cell viability assay, clearly demonstrating the nearly fourfold change in IC_50_ values (Fig. 4k). Importantly, BRPF1 knockdown in parental cells did not alter the IC_50_ value (Supplementary Fig. 5g, h).

To investigate whether the observed phenotype is solely dependent on BRPF1 or extends to the entire MOZ–MORF complex, we performed KOs for all complex members (Supplementary Fig. 5i). We included the members of HBO1 complex because the two complexes have shared members. In contrast to BRPF1, none of the complex members exhibited sensitization Taxol upon KO (Fig. 4l, m). To further confirm the role of BRPF1 on Taxol resistance, we conducted a rescue experiment by reintroducing a PAM mutant version of BRPF1 into the KO background (Fig. 4n and Supplementary Fig. 5j). The ability to form colonies in the presence of Taxol was restored when BRPF1 was reexpressed after the KO (Fig. 4o, p). Overall, these findings suggest that BRPF1 has a critical role in the Taxol resistance of TNBC cells.

To understand the clinical significance of BRPF1 alongside the members of MOZ–MORF and HBO1 complexes, we analyzed publicly available breast cancer patient data from The Cancer Genome Atlas (TCGA), which includes all breast cancer subtypes, such as luminal A, luminal B, Her2 and basal subtype. Our findings indicated that, in the basal subtype containing TNBC, there is a distinct upregulation solely in BRPF1 mRNA levels, in contrast to other members of the MOZ–MORF and HBO1 complexes (Supplementary Fig. 6a). In addition, BRPF1 expression positively correlated with increasing breast cancer grade, further distinguishing it from other complex members (Supplementary Fig. 6b). Extending this analysis to patient outcomes, we observed that high BRPF1 expression is significantly associated with poorer survival in patients who received systemic chemotherapy, suggesting a role for BRPF1 in therapy resistance (Supplementary Fig. 6c)^39^. Together, these findings indicate that BRPF1 may serve as both a prognostic marker and a potential contributor to chemotherapy resistance in patients with breast cancer.

### BRPF1 inhibitors recapitulate the effects of BRPF loss on Taxol-resistant cells

**Fig. 5:**
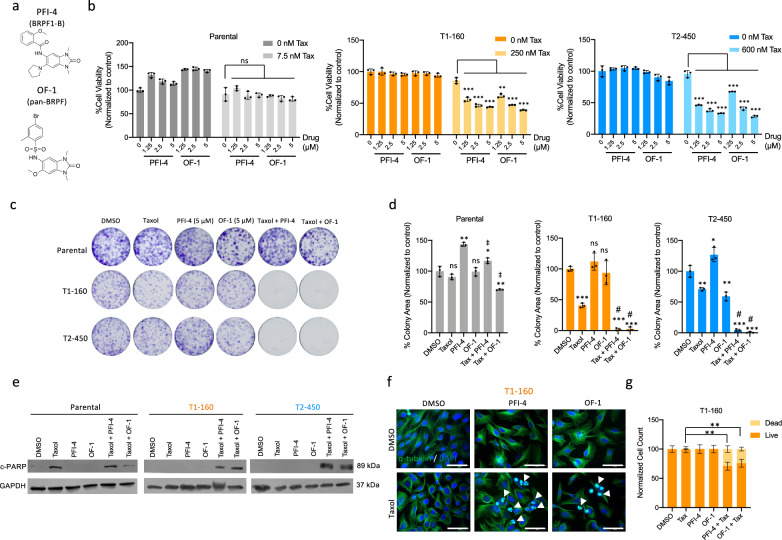
Chemical probe library screens reveal BRPF1 inhibitors as Taxol sensitizers. **a** Chemical structures of BRPF1 inhibitors identified during probe library screen. PFI-4 specifically targets BRPF-1, and OF-1 is a pan-BRPF inhibitor acting against BRPF1–BRPF2–BRPF3. **b** Validations of the effect of PFI-4 and OF-1 in combination with Taxol on parental, T1-160 and T2-450 cells. **c** Clonogenic assay results of Taxol (3 nM for parental, 125 nM for T1-160 and 300 nM for T2-450) and BRPF1i (5 µM each) combinations **d** Quantification of colonies in **c**. *P* values determined by two-tailed Student’s *t*-test in comparison with DMSO; *P < 0.05, **P < 0.01, ***P < 0.001. ‡ indicates *P* < 0.01 and # indicates *P* < 0.001 in comparison with Taxol. **e** Western blot analysis for cleaved-PARP (c-PARP) and GAPDH as loading control upon combination treatment. **f** Immunofluorescence staining with α-tubulin (green) and DAPI (blue) in the presence of Taxol (parental: 160 nM, T1-160: 160 nM, T2-450: 450 nM) and/or BRPF1 inhibitors (5 µM each) for 16 h. **g** Quantification of dead cells in **f**. Scale bar, 50 µm.

Through the unbiased chemical probe library screen (Fig. 3d), several BRPF1 inhibitors demonstrated the potential to sensitize Taxol-resistant cells. Among them, PFI-4 (targets BRPF1b) and OF-1 (targets pan-BRPF) are structurally different (Fig. 5a). These inhibitors significantly reduced cell viability when combined with Taxol in resistant cells, with no impact on parental cells (Fig. 5b). GSK6853 was another BRPF1-specific inhibitor identified as a potential hit; however, it had a low effect on the cell viability of T1-160 and no effect on parental and T2-450 cells (Supplementary Fig. 7a). We also tested the effects of GSK5959, which has a chemical structure similar to that of PFI-4 but was not present in the library. GSK5959 sensitized T1-160 cells to Taxol, and its effect on T2-450 cells was similar to the results observed on KO samples decreasing cell viability even in the absence of Taxol (Supplementary Fig. 7a). For subsequent experiments, we utilized mainly PFI-4 and OF-1 as they demonstrated consistent efficacy in both resistant cell lines and were identified during the initial library screen. Clonogenic assay on T1-160 and T2-450 cells demonstrated that the combination of Taxol with PFI-4 and OF-1 impaired the viability of resistant cells with minimal impact on parental cells (Fig. 5c, d). Bright-field images of Taxol- as well as PFI-4- and OF-1-treated cells also illustrated the abnormal cell shapes and lower number of cells compared with controls (Supplementary Fig. 7b). Increased cleavage of PARP was observed in Taxol-treated parental cells and combination-treated T1-160 and T2-450 cells (Fig. 5e). Immunofluorescence staining of α-tubulin on T1-160 cells in the presence of Taxol and BRPF1 inhibitors showed round cells with shrunken microtubules (Fig. 5f, g).

Although KO of MOZ–MORF complex members did not affect cell viability, we evaluated KAT6A and KAT6B inhibitors for potential effects on Taxol resistance (Supplementary Fig. 7c). While KAT6A inhibition alone with WM-1119 had no significant effect on cell viability when combined with Taxol, WM-8014 (KAT6A and KAT6B inhibitor) showed a slight decrease. However, the efficacy of KAT6A and KAT6B inhibitors did not match that of PFI-4 and OF-1, suggesting that the reader function of BRPF1 may be more crucial than its scaffolding function for the HAT activity of the complex. In summary, our findings indicate that BRPF1 inhibitors, when combined with Taxol, significantly decrease cell viability in resistant cells.

### Transcriptomic changes caused by BRPF1 reveal defects in translation machinery in Taxol-resistant cells

**Fig. 6:**
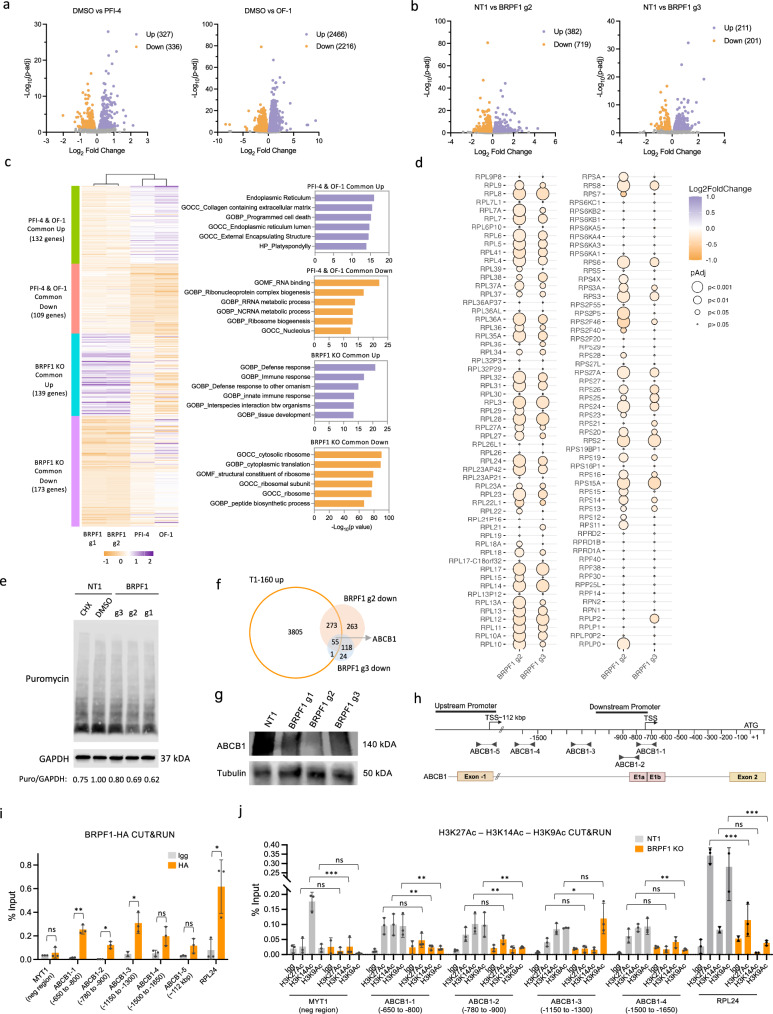
Transcriptomic changes caused by BRPF1 inhibition indicate ribosome biogenesis defects. **a** Volcano plots showing differentially expressed genes (*P* < 0.05) after 72 h of PFI-4 (5 µM) (left) and OF-1 (5 µM) (right) treatment on T1-160 cells. **b** Volcano plots showing differentially expressed genes (*P* < 0.05) upon BRPF1 KO with sgRNA #2 and #3 compared with nontargeting control (NT1) on T1-160 cells. **c** A heatmap showing the mRNA expression levels of all genes that were commonly downregulated or upregulated in BRPF1 inhibitor-treated or sgRNA-infected cells. On the right, significant pathways of the common upregulated and downregulated genes are demonstrated by the overlap analysis via MsigDB. **d** A balloon plot depicting the LFCs and adjusted *P* values of ribosome-related genes in BRPF1-KO cells. **e** SUnSET assay showing global translation levels in control and BRPF1-KO cells. Band intensities were normalized to the loading control GAPDH, and the ratio of NT1 sample was normalized to 1. **f** A comparison of the genes upregulated in T1-160 cells and downregulated in BRPF1-KO T1-160 cells. **g** Western blot showing ABCB1 protein levels in BRPF1-KO T1-160 cells. **h** The *ABCB1* gene structure with the locations of the primers used in the CUT&RUN-qPCR experiment. **i**, CUT&RUN-qPCR results showing the enrichment of BRPF1 binding sites on *ABCB1* and *RPL24* promoters in T1-160 cells expressing HA-tagged BRPF1. *P* values determined by two-tailed Student’s *t*-test by comparing each HA group with its IgG control; *P < 0.05, **P < 0.01, ***P < 0.001. **j**, CUT&RUN-qPCR results showing the decrease in the enrichments of H3K27Ac, H3K14Ac and H3K9Ac histone marks on *ABCB1* and *RPL24* promoters upon BRPF1 loss. Data are representative of three independent biological replicates. *P* values determined by two-way ANOVA with Tukey’s post-hoc test; *P < 0.05, **P < 0.01, ***P < 0.001.

To gain insight into the transcriptomic changes caused by BRPF1, we treated T1-160 cells with PFI-4 and OF-1 or knocked out BRPF1 and performed RNA sequencing. PFI-4 caused the upregulation of 327 genes and downregulation of 336 genes, while OF-1 had a greater impact on the transcriptome, with over 2,000 genes being either upregulated or downregulated (Fig. 6a). BRPF1 targeting sgRNA #2 resulted in 382 upregulated and 719 downregulated genes. sgRNA #3 caused the upregulation and downregulation of approximately 200 genes (Fig. 6b). We then performed overlap analysis with biological processes from the Molecular Signature Database (MsigDB) on commonly downregulated and upregulated genes upon inhibitor or sgRNA treatment (Fig. 6c). Programmed cell death was one of the signatures upregulated upon inhibitor treatment. Notably, commonly downregulated genes significantly overlapped with numerous RNA and ribosome biogenesis-related pathways. The majority of the downregulated genes in BRPF1 sgRNA #3 were common with sgRNA #2, and these genes significantly overlapped with ribosome biogenesis- and translation-related pathways (Fig. 6c). As a result, inhibition or KO of BRPF1 significantly decreased the expression of ribosome biogenesis and translation-related genes, suggesting an impairment of translation machinery. We observed a slight but consistent decrease in the mRNA levels of nearly all ribosome-related genes upon BRPF1 KO (Fig. 6d and Supplementary Fig. 8a) and inhibition (Supplementary Fig. 8b). To assess the functional role of downregulation of ribosome-related genes, we measured the global translation rate with the SUnSET assay^31^ (Fig. 6e). Notably, all BRPF1-KO T1-160 cells had a lesser amount of puromycin-labeled proteins compared with the control, suggesting that the translation rate is decreased upon BRPF1 loss.

Next, we focused on the set of genes upregulated in resistant cells, that were downregulated upon BRPF1 KO (Fig. 6f). Notably, *ABCB1* was among this set of genes. ABCB1 protein was expressed at significantly lower levels in BRPF1 KO-resistant cells (Fig. 6g). In addition, we observed significant downregulation of the ABCB1 expression in Taxol-resistant cells treated with either BRPF1 inhibitors or siRNA (Supplementary Fig. 8c). To assess the clinical relevance of this relationship, we analyzed publicly available datasets of patients with metastatic breast cancer who had received chemotherapy and observed a subtle but statistically significant positive correlation between BRPF1 and ABCB1 expression (Supplementary Fig. 8d). These results suggest a potential link between BRPF1 and ABCB1-mediated drug resistance.

We investigated whether BRPF1 directly binds to the *ABCB1* promoter to facilitate its transcription and performed a CUT&RUN assay followed by qPCR using anti-HA antibody to pull down HA-tagged BRPF1. Our results demonstrated a significant enrichment of BRPF1 at the *ABCB1* promoter, specifically at the downstream promoter region near the transcription start site (TSS), rather than the upstream promoter, establishing a mechanistic link between BRPF1 and ABCB1-mediated drug resistance (Fig. 6h, i)^40^. In addition, as a proof of concept, we confirmed BRPF1 binding at the promoter of the ribosomal protein gene *RPL24*, reinforcing its broader function in transcriptional regulation consistent with the RNA sequencing results and functional assays (Fig. 6i). To further validate BRPF1’s role in ABCB1 transcriptional regulation, we performed a CRISPR interference experiment targeting the BRPF1 binding sites on the *ABCB1* promoter (Supplementary Fig. 8e). Notably, sgRNAs targeting the TSS and ATG proximal regions of ABCB1 markedly reversed Taxol resistance, further displaying the requirement of BRPF1 binding around the TSS region to maintain the resistant state. These findings support our CUT&RUN-qPCR results, which demonstrated BRPF1 binding at regions surrounding the TSS.

To investigate histone acetylation changes at BRPF1 target genes, we performed CUT&RUN-qPCR on NT versus BRPF1-KO T1-160 cells (Fig. 6j). Our results demonstrated a significant reduction in the enrichment of H3K14Ac and H3K9Ac—histone marks specifically recognized by BRPF1—at the promoters of *ABCB1* and *RPL24* upon BRPF1 KO. Interestingly, a decrease in H3K27Ac levels, an open chromatin mark, upon BRPF1 loss was strongly evident in the *RPL24* promoter, suggesting that BRPF1 regulates the transcription of ribosomal protein genes (Fig. 6j). PFI-4 treatment on T1-160 cells similarly caused a reduction in H3K14Ac levels on the *ABCB1* promoter, while global H3K14Ac levels remained unaffected, suggesting a locus-specific regulation by BRPF1 (Supplementary Fig. 8f, g).

## Discussion

The present study aimed to unravel the epigenetic mechanisms underlying Taxol resistance in TNBC cells. To recapitulate chemotherapy resistance in vitro, two independent Taxol-resistant TNBC cell lines (T1-160 and T2-450) were generated through a dose-escalation method. These cells demonstrated characteristics typical of chemotherapy resistance, including increased IC_50_ values, slower growth, altered cell morphology and resistance to apoptosis induction compared with parental cells. Transcriptomic analyses revealed distinct characteristics in each resistant line, such as decreased oxidative phosphorylation in T1-160^41–43^ and upregulation of inflammatory response-related pathways in T2-450^44^. Notably, both cell lines exhibited elevated expression of ABCB1, an ATP-binding cassette transporter associated with MDR, which was further validated through functional assays emphasizing the role of ABCB1 in our Taxol-resistant TNBC cell lines.

Despite successfully reversing resistance in T1-160 cells using various ABCB1 inhibitors, including verapamil, elacridar and zosuquidar, the high toxicity and limited benefits of these inhibitors preclude their use in combination therapies. CNV is known to contribute to drug resistance; however, recent studies indicated that, although the copy number of *ABCB1* was elevated, it was insufficient to activate *ABCB1* expression without the transcriptional regulation, emphasizing the inadequacy of CNV alone^13,20,28,45^. Thus, understanding the upstream regulators of ABCB1 specific to cancer cells is crucial for developing effective and safe treatment strategies.

To explore potential epigenetic regulators contributing to Taxol resistance, comprehensive epigenome-wide CRISPR–Cas9 and epigenetic probe library screens were used in this study. The convergence of results from both screens pinpointed BRPF1 as a key candidate. Although the prioritization of BRPF1 for further investigation appears to stem from the epigenetic probe library screen, the CRISPR screen offers additional insights into the long-term consequences of gene loss, enabling us to assess whether compensatory mechanisms or adaptive responses are at play. Notably, several genes identified in our CRISPR screen, including *ARID1A*, *PSIP1*, *RNF2*, *UBE2D* and *MEN1*, were previously associated with chemoresistance in various cancer types, reinforcing the biological relevance of our findings^46–51^. Although the depletion effects in CRISPR screens may appear modest, they often reflect sustained functional dependencies rather than acute responses, making them valuable indicators of therapeutic vulnerabilities. This highlights the significance of BRPF1 and other candidate genes as promising targets for further investigation in the context of Taxol resistance.

BRPF1 orchestrates histone acetylation by bringing the MEAF6 and ING5 accessory proteins and MOZ–MORF catalytic subunits together. It is known that BRPF1 is indispensable during embryonic development^52,53^ and causes intellectual disability when mutated^54^. In the context of cancer, upregulation of BRPF1 is associated with low survival rates in patients with hepatocellular carcinoma^55^. Analysis of TCGA data revealed BRPF1 mutations or CNVs in various cancer types^35,56^. However, the link between BRPF1 and chemoresistance was largely unexplored. BRPF2 and BRPF3, close paralogs of BRPF1, may also form complexes with MOZ–MORF. BRPF1 KO significantly impaired colony-forming abilities in Taxol-resistant cells in the presence of Taxol, surpassing the impact of BRPF2 or BRPF3 KOs. In T2-450 cells, BRPF1 loss impaired the colony-forming ability even in the absence of Taxol, suggesting that BRPF1 was required for cell fitness of Taxol-resistant T2-450 cells. Intriguingly, the catalytic or accessory subunits of the MOZ/MORF complex showed no effect, emphasizing the unique role of BRPF1 as a reader in the chemoresistance context. Indeed, the rescue of BRPF1 KO by overexpressing the PAM mutant version of BRPF1 was sufficient to restore Taxol resistance of T1-160 cells.

We investigated BRPF1 inhibitors, PFI-4 and OF-1, revealing consistent efficacy in reducing cell viability when combined with Taxol, suggesting their potential as future therapeutic agents. Transcriptomic changes induced by BRPF1 loss or inhibition provided valuable insights into defects in Taxol-resistant cells, particularly the downregulation of ribosomal and translation-related genes. Functional assays further demonstrated a decreased global translation rate in BRPF1-KO cells, linking BRPF1 to the regulation of the translation machinery in Taxol-resistant TNBC cell lines.

RUNX2, a downstream target of BRPF1, has been reported to negatively regulate the transcriptional control of rRNA genes^57,58^. The deficiency of another BRPF1 target, RUNX1, decreases ribosome biogenesis and translation, causing hematopoietic stem and progenitor cells to develop resistance to genotoxic stress^59^. The discrepancy in the effects of RUNX transcription factors (TFs) might result from the chromatin regulatory factors with which they associate^60^. It has been reported that MOZ and MORF function as transcriptional coactivators for RUNX TFs^61^. Although BRPF1 was not previously associated with ribosome biogenesis, our findings suggest that BRPF1 regulates the translation machinery by directly binding to the promoters of ribosomal protein genes, such as *RPL24*, in Taxol-resistant TNBC cell lines. It is possible that BRPF1, as the scaffolding member of the complex that directs it to specific genomic locations, might be regulating the interaction between MOZ/MORF and RUNX TFs in the chemoresistance context.

In this study, through genetic and chemical screens, we identified BRPF1 as a critical regulator of Taxol resistance in TNBC cells. The depletion of BRPF1 through CRISPR–Cas9 or siRNA, as well as inhibition using PFI-4 or OF-1, resulted in a reduction of ABCB1 expression. The regulation of ABCB1 levels by BRPF1 involved its direct binding to the *ABCB1* promoter surrounding the TSS region in the downstream promoter. Analysis of publicly available breast cancer patient data also revealed elevated expression of BRPF1 in the basal subtype as well as in higher-grade tumors, indicating a role for BRPF1 in disease progression. Moreover, high BRPF1 expression was significantly associated with poorer survival in patients with breast cancer who received systemic chemotherapy. Similarly, in an independent study, we demonstrated that castration-resistant prostate cancer cells also depend on BRPF1 in docetaxel and cabazitaxel resistance, highlighting BRPF1’s role as an *ABCB1* regulator controlling mTOR and unfolded protein response (UPR) signaling^62^. Another recent study showed that BRPF1 associates with ERα and targeting BRPF1 reduces cell proliferation in endocrine-therapy-resistant breast cancers^63^, suggesting its role in regulating therapy resistance.

**Fig. 7:**
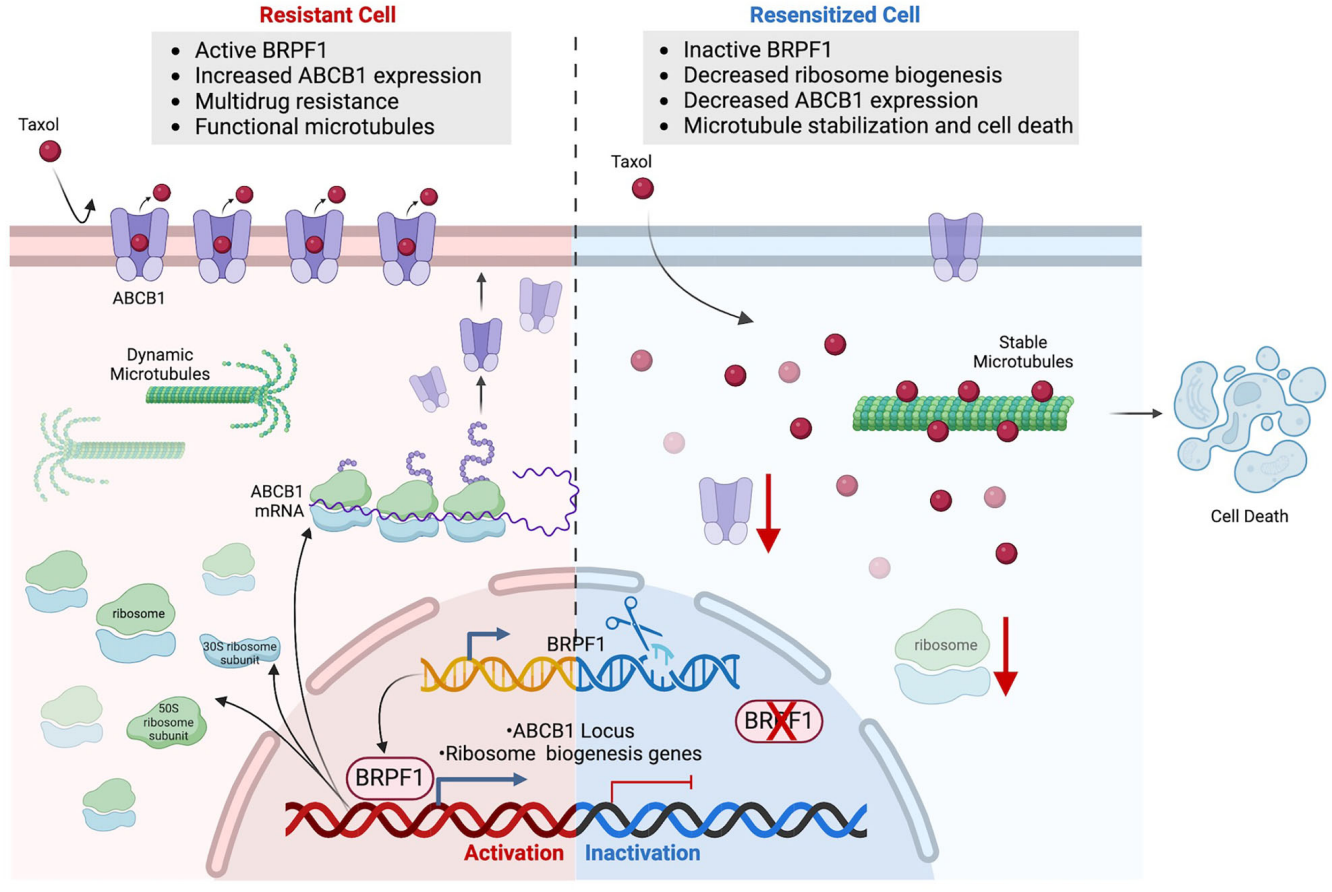
Proposed model of Taxol resistance regulation by BRPF1. The role of BRPF1 in Taxol resistance is summarized. Created with BioRender.com.

In this study, we revealed that BRPF1 deficiency led to diminished ribosome biogenesis, causing a decline in the global translation rate, potentially contributing to an overall decrease in ABCB1 levels in resistant cells (Fig. 7). The intricate interplay between BRPF1, ribosome biogenesis, and ABCB1 expression elucidates a novel mechanism underlying Taxol resistance in TNBC cells and holds promise as a therapeutic strategy, particularly in patients exhibiting elevated ABCB1 levels.

## Supplementary information

Supplementary Information

## Data Availability

EPIKOL screen and RNA sequencing data are deposited to the NCBI GEO database with the accession numbers GSE262577 and GSE262353.
