# Supplementary material for: Chromatin-focused genetic and chemical screens identify BRPF1 as a targetable vulnerability in Taxol-resistant triple-negative breast cancer
Source: Exp Mol Med. 2025 Jun 30;57(6):1294–307. doi: 10.1038/s12276-025-01466-5 (PMC12229664; doi:10.1038/s12276-025-01466-5)
Supplement: Supplementary file 1 — Supplementary Information [file 12276_2025_1466_MOESM1_ESM.pdf]

SUPPLEMENTARY INFORMATION

SUPPLEMENTARY MATERIALS AND METHODS

Colony formation assay. Cells were seeded as 1000 cells/well for parental and 1500 cells/well for resistant cells in triplicate in 6-well plates. For sgRNA-containing experiments, cells were seeded after puromycin selection. Next day, drug treatments were performed as indicated in each experiment. 72 hours later, medium was refreshed, and cells were allowed to grow for 10-14 days. Fixation and staining of the cells were performed as described[1]. Quantification of the area occupied by colonies was performed by using ImageJ[2].

Live Cell Microscopy. Cells were seeded as 75.000 cells/well in 12-well plates and next day treated with Taxol; 160 nM for Parental and T1-160 and 450 nM for T2-450. For drug combination experiments, concentrations of PFI-4 and OF-1 was 5 µM. After drug treatment, phase contrast images were taken as 2x2 with 10x objectives using Cytation5 (Biotek, USA) every 15 minutes for 72 hours.

Fluorescent-labeled resistant cell assay. Parental cells were transduced with PGK-H2BeGFP (Addgene #21210) viruses, resistant cells were transduced with PGK-H2BmCherry (Addgene #21217) at MOI~5 ensure each cell was fluorescently labeled. Parental and resistant cells were mixed in a 1:1 ratio as 40.000 cells in 24-well plates. Next day, parental:T1-160 mixtures were treated with 160 nM while parental:T2-450 mixtures were treated with 450 nM Taxol. Phase contrast, GFP and Texas Red images were taken as 2x2 with 10x objective using Cytation5 (BioTek, USA) in every 15 minutes for 72 hours. Number of mCherry+ and eGFP+ cells were counted from images using Gen5 software (BioTek, USA).

Annexin V staining. Annexin V staining was performed as previously described[1]. Parental and T1-160 cells were treated with 160 nM, T2-450 cells were treated with 450 nM Taxol. Cells were collected 24 hours after treatment.

Western blotting. Western blotting was performed as described[1]. Primary antibodies used in this study are listed in **Supplementary Table 1**.

Cell cycle analysis. Parental and T1-160 cells were treated with 160 nM, T2-450 cells were treated with 450 nM Taxol. $1x10^6$ cells were collected 8 hours after Taxol treatment and cell cycle analysis was performed as described earlier[1].

RNA sequencing and transcriptome analysis. RNA isolation, library preparation, sequencing and bioinformatic analysis were performed as described[1]. For parental-resistant comparisons, differentially expressed genes (DEGs) were defined with a threshold for $Log_2FoldChange>2$ (up-regulated) or $Log_2FoldChange<-2$ (down-regulated) and $p<0.001$. For drug or sgRNA-treated T1-160 cells, DEGs were determined as the genes that have padj<0.05. Gene set enrichment analysis was performed with $Log_2FoldChange$ rank-ordered gene lists by using GSEA software and all available gene sets from MsigDB at the date of January 24th, 2022[3].

Quantitative RT-PCR. RNA isolation and cDNA synthesis were performed as described[1]. List of qPCR primers can be found in **Supplementary Table 2**.

Copy Number Variation analysis. Genomic DNAs of parental and resistant cell lines were isolated by using MN Nucleospin Tissue kit according to manufacturer's recommendation. 10 ng of genomic DNA was used as template for qPCR using SYBR Green, as per standard procedures. Primers used in copy number variation analysis are listed in **Supplementary Table 6.**

Calcein assay. Calcein-AM is a membrane permeable dye that is converted to a fluorescent calcein by intracellular esterases. It is also a substrate for P-Glycoprotein (Pgp, ABCB1). Increased Pgp expression enhances the efflux of Calcein-AM and decreases fluorescence signal. Cells were seeded as 65 000 cells/well in 24-well plate. Next day, cells were treated with 10 µM Verapamil for 30 minutes at 37°C. Calcein-AM (C1430, Invitrogen) was added in final concentration of 125 nM and cells were incubated for another 30 minutes at 37°C. Images were taken with phase contrast and GFP filters as 2x2 with 10x objective using Cytation5 (BioTek, USA). Quantification of green fluorescence was performed in Synergy H1 Plate Reader (BioTek, USA).

Analysis of breast cancer patient data. BRPF1 expression analysis across breast cancer subtypes and histological grades was conducted using TCGA-PanCancer Atlas[4] and METABRIC datasets[5,6], accessed via cBioPortal[7]. Statistical analysis using One-way ANOVA was performed with Prism 8 (GraphPad Software, Inc.). Survival analysis of patients who received systemic chemotherapy was performed using KMPlotter[8]. Patients were stratified into high- and low-expression groups based on the upper quartile of BRPF1 expression. Correlation analysis of BRPF1 and ABCB1 expression was also performed using Metastatic Breast Cancer dataset in CBioPortal[7].

CRISPR inhibition. sgRNAs targeting different regions of ABCB1 promoter, determined according to the results of the CUT&RUN experiments, were cloned into LentiGuide-Puro (Addgene, #52963). List of sgRNAs can be found on **Supplementary Table 7**. T1-160 cells were infected with Lenti-dCas9-KRAB-blast (Addgene, #89567) and selected with blasticidin. Later cells were infected with Nontargeting control or sgRNAs targeting ABCB1 locus, on PT5, 1500 cells/well were seeded into 6-well plates for colony formation assay.

REFERENCES

1 Yedier-Bayram, O. *et al.* EPIKOL, a chromatin-focused CRISPR/Cas9-based screening platform, to identify cancer-specific epigenetic vulnerabilities. *Cell Death Dis* **13**, 710 (2022).

2 Schneider, C. A., Rasband, W. S. & Eliceiri, K. W. NIH Image to ImageJ: 25 years of image analysis. *Nat Methods* **9**, 671-675 (2012).

3 Subramanian, A., Kuehn, H., Gould, J., Tamayo, P. & Mesirov, J. P. GSEA-P: a desktop application for Gene Set Enrichment Analysis. *Bioinformatics* **23**, 3251-3253 (2007).

4 Berger, A. C. *et al.* A Comprehensive Pan-Cancer Molecular Study of Gynecologic and Breast Cancers. *Cancer Cell* **33**, 690-705 e699 (2018).

5 Curtis, C. *et al.* The genomic and transcriptomic architecture of 2,000 breast tumours reveals novel subgroups. *Nature* **486**, 346-352 (2012).

6 Pereira, B. *et al.* The somatic mutation profiles of 2,433 breast cancers refines their genomic and transcriptomic landscapes. *Nat Commun* **7**, 11479 (2016).

7 Cerami, E. *et al.* The cBio cancer genomics portal: an open platform for exploring multidimensional cancer genomics data. *Cancer Discov* **2**, 401-404 (2012).

8 Gyorffy, B. Survival analysis across the entire transcriptome identifies biomarkers with the highest prognostic power in breast cancer. *Comput Struct Biotechnol J* **19**, 4101-4109 (2021).

SUPPLEMENTARY FIGURES

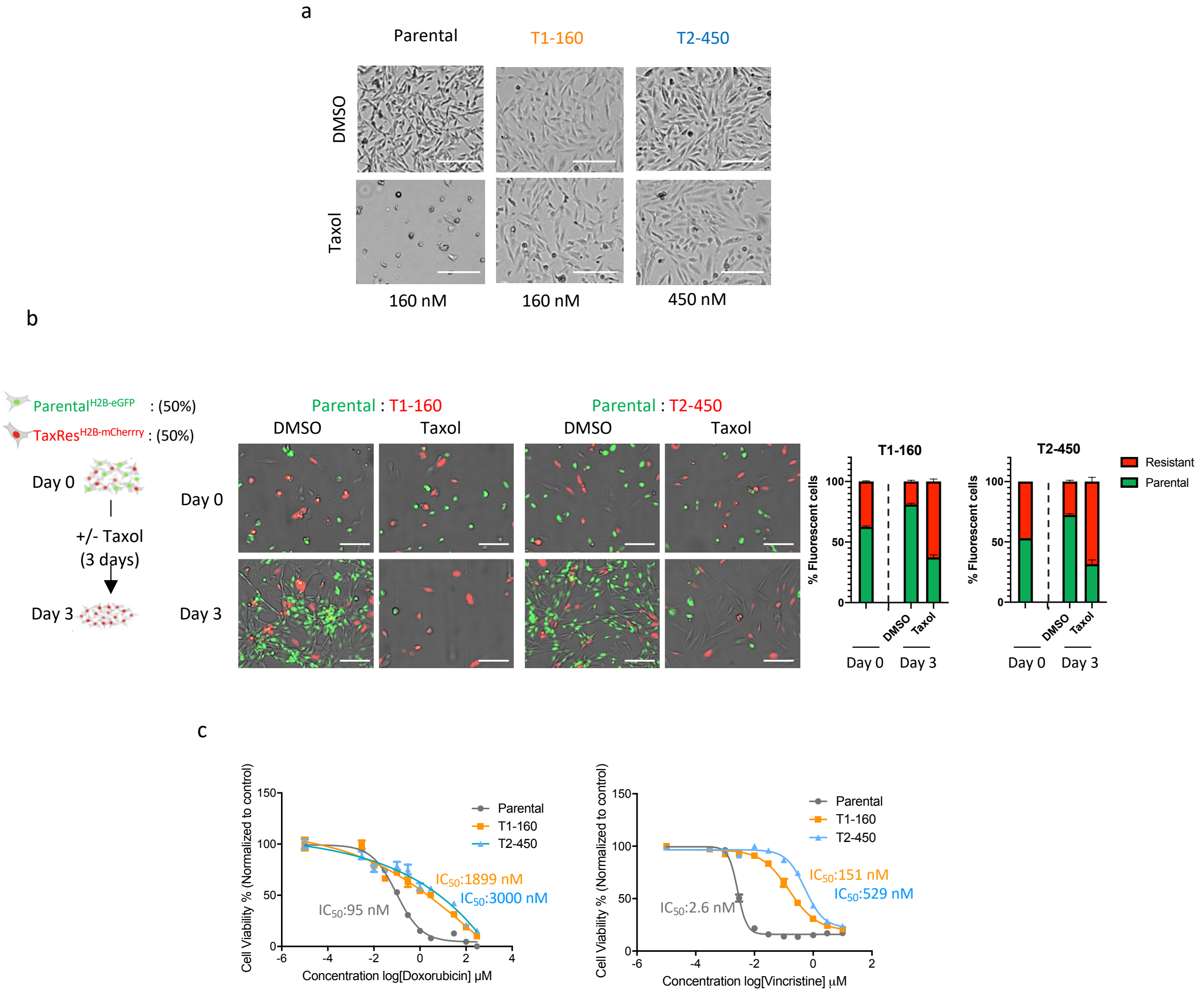

Supplementary Figure 1. Taxol-resistant phenotype. a. Representative images from live cell imaging. Cells were seeded to 12-well plates in the presence or absence of Taxol (Parental: 160 nM, T1-160: 160 nM, T2-450: 450 nM) and images were taken at every 15 minutes for 3 days after drug treatment. Scale bar: 200 μm**. b.** Competition assay in the presence of Taxol. Parental cells were labeled with PGK-H2B-eGFP virus, resistant cells were labeled with pGK-H2B-mCherry and mixed in 1:1 ratio. After +/- Taxol treatment, images were taken at every 20 minutes for 3 days after drug treatment. Scale bar: 100 μm. **c.** Cross-resistance phenotype of T1-160 and T2-450 cells upon Doxorubicin and Vincristine treatment.

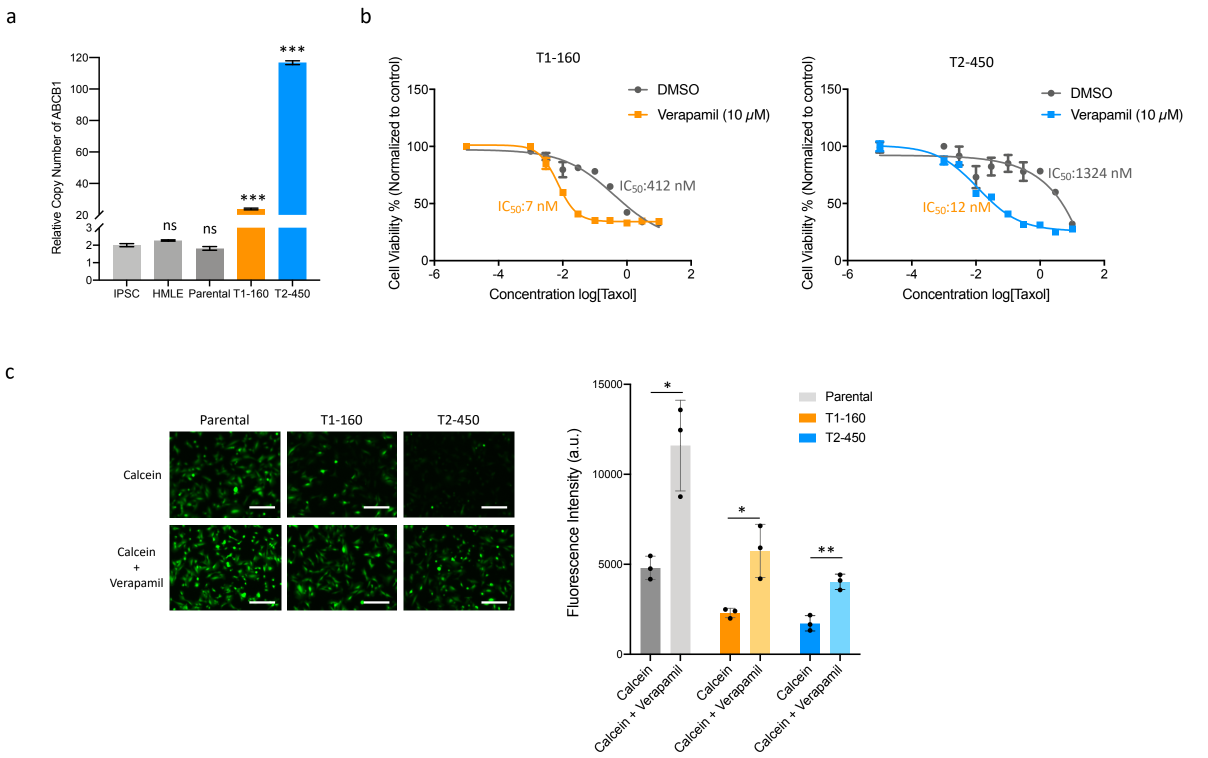

Supplementary Figure 2. ABCB1 expression and function in Taxol-resistant cells. a. Copy number variation (CNV) analysis of ABCB1 in Taxol-resistant cells when compared to control cell lines (HMLE and IPSC) with normal karyotypes. **b.** Effect of verapamil on cell viability of Taxol-resistant T1-160 (left) and T2-450 cells (right). **c.** Representative images of Calcein-AM (125 nM) assay in the presence of verapamil (10 µM) on parental and resistant cells. Scale bar: 100 µm. Quantification of fluorescent signal was performed using microplate reader. P values determined by two-tailed Student's t-test in comparison to control group; *$p < 0.05$, **$p < 0.01$, ***$p < 0.001$.

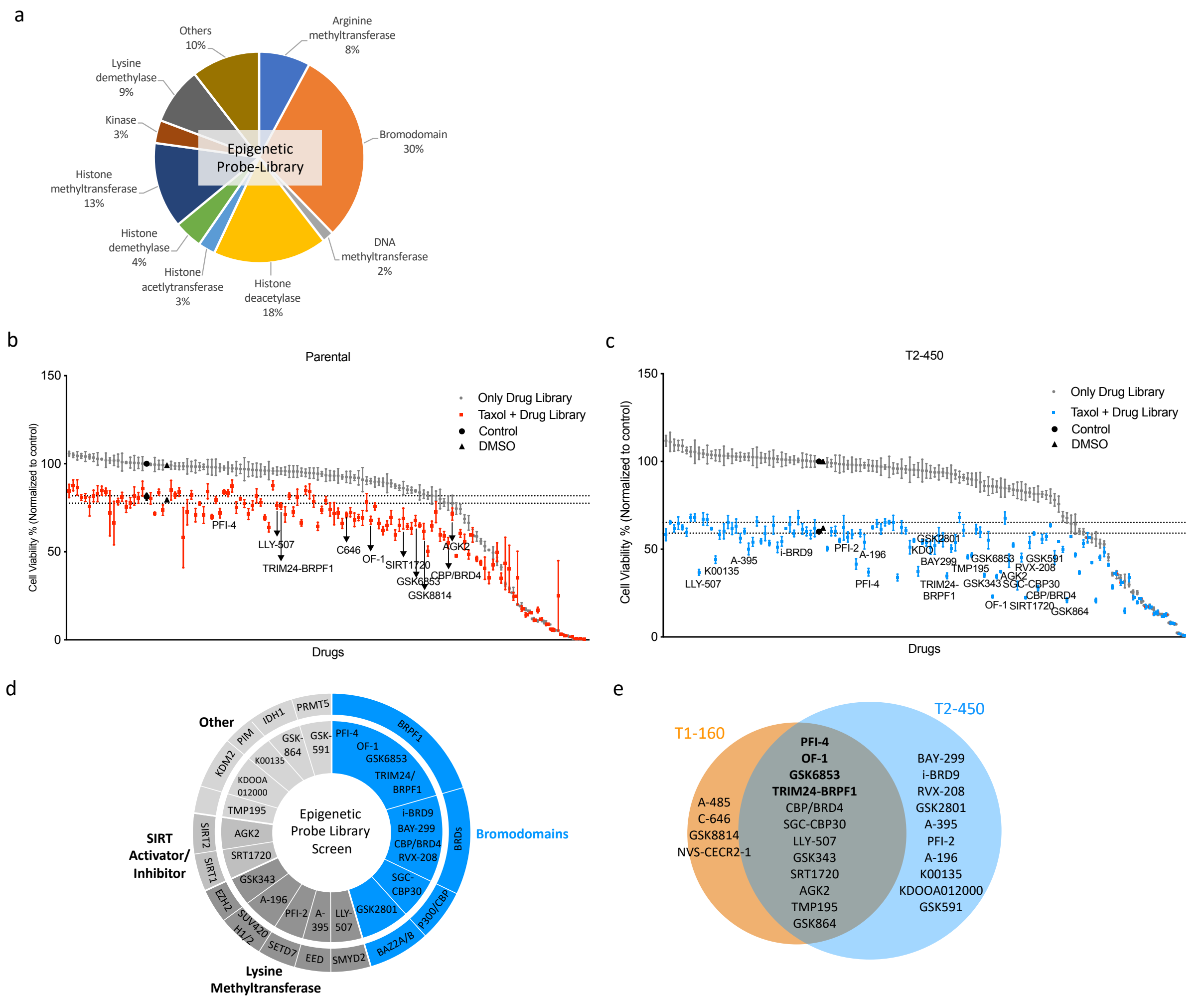

Supplementary Figure 3. Epigenetic probe library screen on parental and T2-450 cells. **a.** Pie chart showing the epigenetic modifier classes targeted by the epigenetic chemical probe library. **b.** Results of epigenetic probe library screen performed on SUM159PT parental cells. **c.** Results of epigenetic probe library screen performed on T2-450 cells. Both in (B.) and (C.) grey dots represent the effect of epigenetic probes alone while the colored dots show changes in cell viability when Taxol was combined with a specific epigenetic probe. Epigenetic probes that significantly reversed drug resistance are labeled on the T2-450 cell graph. If the inactive compound of a given probe also showed a significant depletion in cell viability, neither of the probes was labeled on the graph. In Parental cell screen results, the epigenetic probes identified as sensitizers in Taxol-resistant cells were labeled **d.** Classification of hits identified in epigenetic probe library screen of T2-450 cells. Target gene of the chemical probes were indicated in the outer circle. **e.** Venn diagram illustrating the common probes with significant effects in T1-160 and T2-450 epigenetic probe library screens.

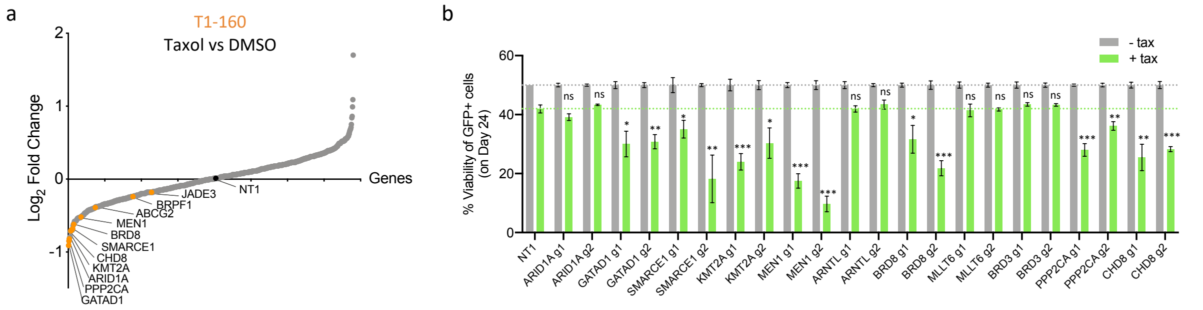

Supplementary Figure 4. Competition assay results of EPIKOL screen on T1-160 cells **a.** Waterfall plot of EPIKOL screen results showing Log_2FoldChanges of genes used in dual-color competition assay **b.** Dual-color competition assay results of candidate genes from T1-160 EPIKOL screen. Gray bars represent the eGFP+ cells in eGFP:mCherry mixtures in the absence of Taxol. Green bars indicate the percentage of eGFP+ cells carrying sgRNA of interest in the population in the presence of Taxol. Untreated sgRNA mixtures were normalized to 50%. P values determined by two-tailed Student's t-test in comparison to control group; ns >0.05, *p < 0.05.

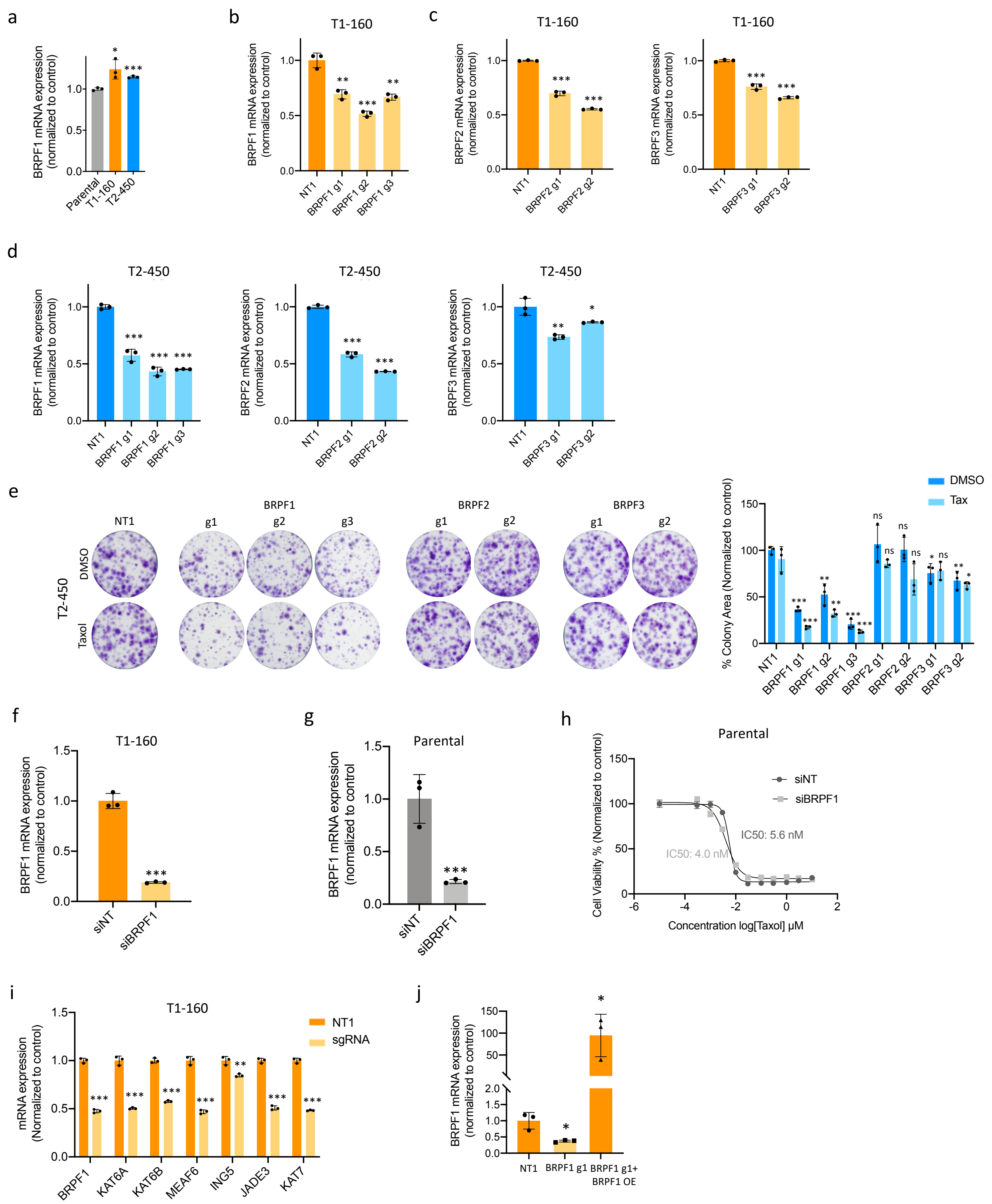

Supplementary Figure 5. Effects of BRPF1, BRPF2, BRPF3 knockout on Parental and Taxol-resistant cells. a. BRPF1 mRNA expression levels in parental and resistant cells. **b.** BRPF1 expression in mRNA level upon BRPF1 knockout in T1-160 cells. **c.** BRPF2 and BRPF3 mRNA expression levels upon their knockouts in T1-160 cells. **d.** BRPF1, BRPF2 and BRPF3 mRNA expression levels upon their knockouts on T2-450 cells **e.** Clonogenic assay showing the effect of BRPF1, BRPF2, and BRPF3 knockouts in the presence of Taxol and quantification of the colonies. **f.** BRPF1 mRNA levels upon BRPF1 siRNA transfection in T1-

160 cells. **g.** BRPF1 mRNA levels upon BRPF1 siRNA transfection in Parental cells. **h.** Cell viability measurement performed with siNT and siBRPF1 samples in the presence of Taxol on Parental cells. **i.** mRNA expression levels of MOZ/MORF and HBO1 complex members upon knockout in T1-160 cells. **j.** BRPF1 mRNA levels upon knockout and overexpression of PAM mutant version of BRPF1 in T1-160 cells. P values determined by two-tailed Student's t-test in comparison to control group; $^{*}p < 0.05$, $^{**}p < 0.01$, $^{***}p < 0.001$.

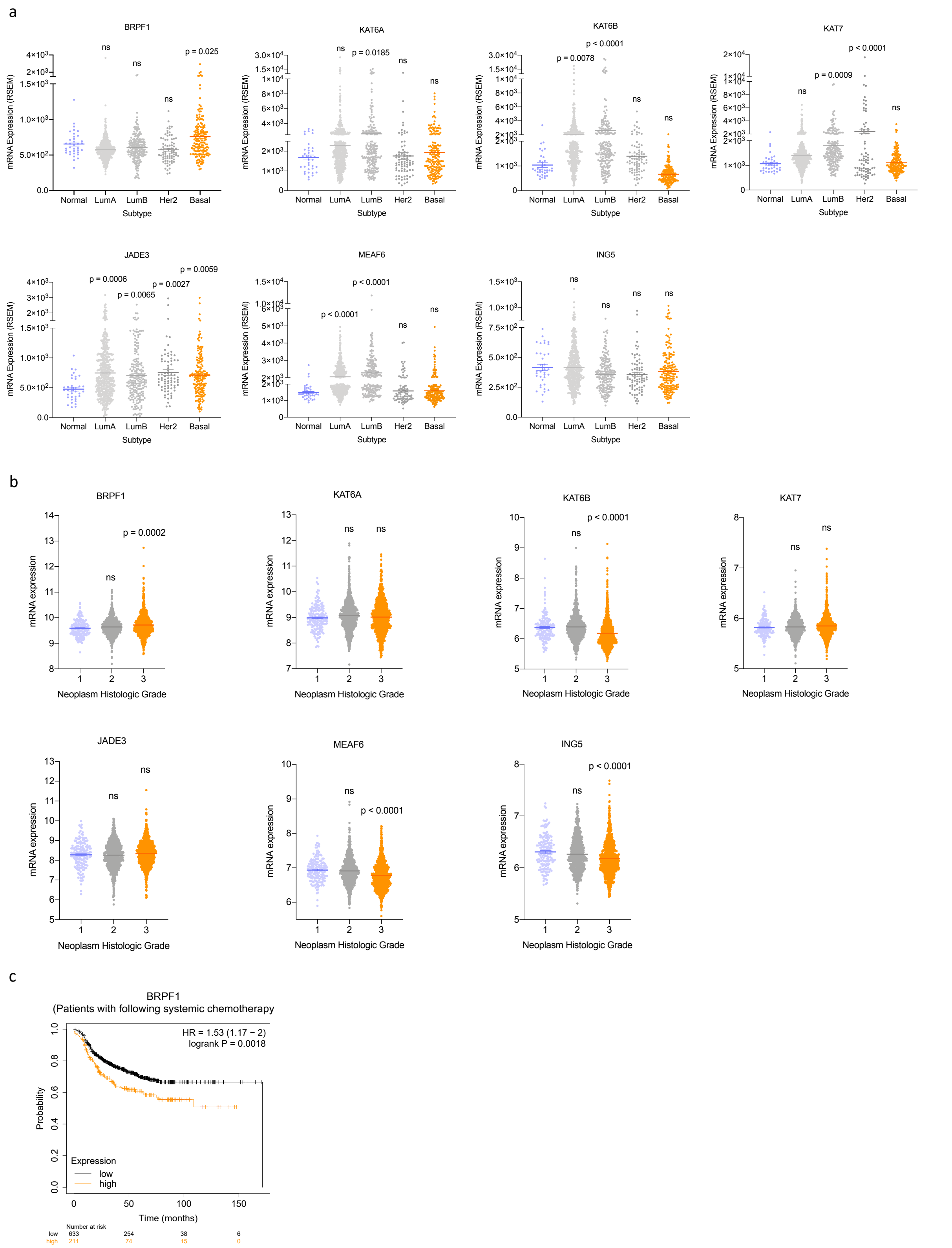

Supplementary Figure 6. MOZ/MORF and HBO1 complex members' expression analysis in breast cancer subtypes, histological grades and patient survival. mRNA levels of MOZ/MORF and HBO1 complex members in clinical samples as accessed through cBioPortal. **a.** Relationship between the expression of the gene and subtype as analyzed using TCGA - Breast Invasive Carcinoma - PanCancer Atlas. Ordinary one-way Anova was performed by

comparing the groups to Normal subtype and p values were written above the group if it is $p < 0.05$ **b.** Histological grade analysis performed using METABRIC database of breast cancer. Ordinary one-way Anova was performed by comparing the higher histological grades to grade 1 and p values were written above the group if it is $p < 0.05$. **c.** Kaplan-Meier plot showing the Relapse Free Survival (RFS) of the breast cancer patients who received systemic chemotherapy, stratified by BRPF1 expression levels. Patients were divided into high- and low-expression groups based on the upper quartile of BRPF1 expression. Survival analysis was performed using KMPlotter. Statistical significance was determined using the log-rank test.

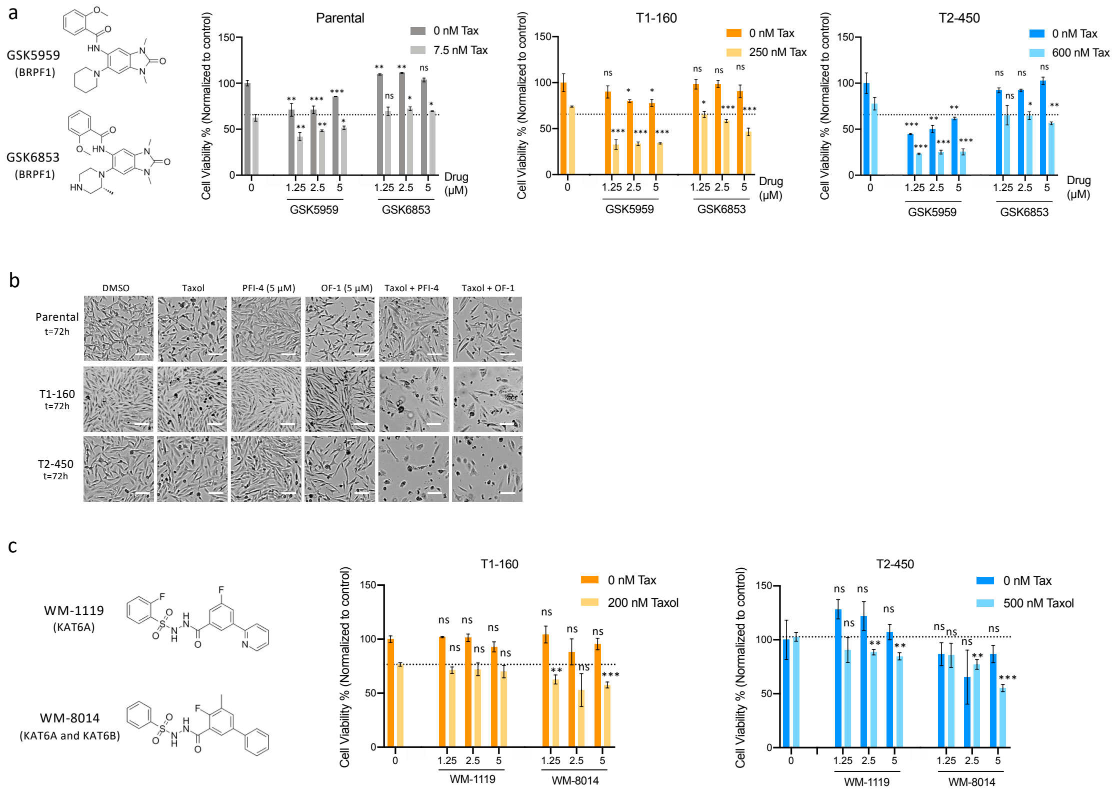

Supplementary Figure 7. BRPF1 and MOZ/MORF inhibitor effects on parental and Taxol-resistant cells. a. (Left) Chemical structures of BRPF1 inhibitors GSK5959 and GSK6853. GSK5959 is an additional specifically inhibitor of BRPF-1, but it was not present in epigenetic probe library. GSK6853 is another BRPF1 inhibitor identified from epigenetic probe library screens in both Taxol resistant cells. (Right) Cell viability results of combination of GSK5959 and GSK6853 with Taxol on Parental and Taxol-resistant cell. **b.** Representative images from live-cell imaging in the presence of PFI-4, OF-1 and Taxol on Parental and Taxol-resistant cells. Scale bar: 100 μm **c.** (Left) Chemical structures of KAT6A/B inhibitors and (Right) cell viability results of their combination with Taxol in T1-160 and T2-450 cells. P values determined by two-tailed Student's t-test in comparison to control group; *$p < 0.05$, **$p < 0.01$, ***$p < 0.001$.

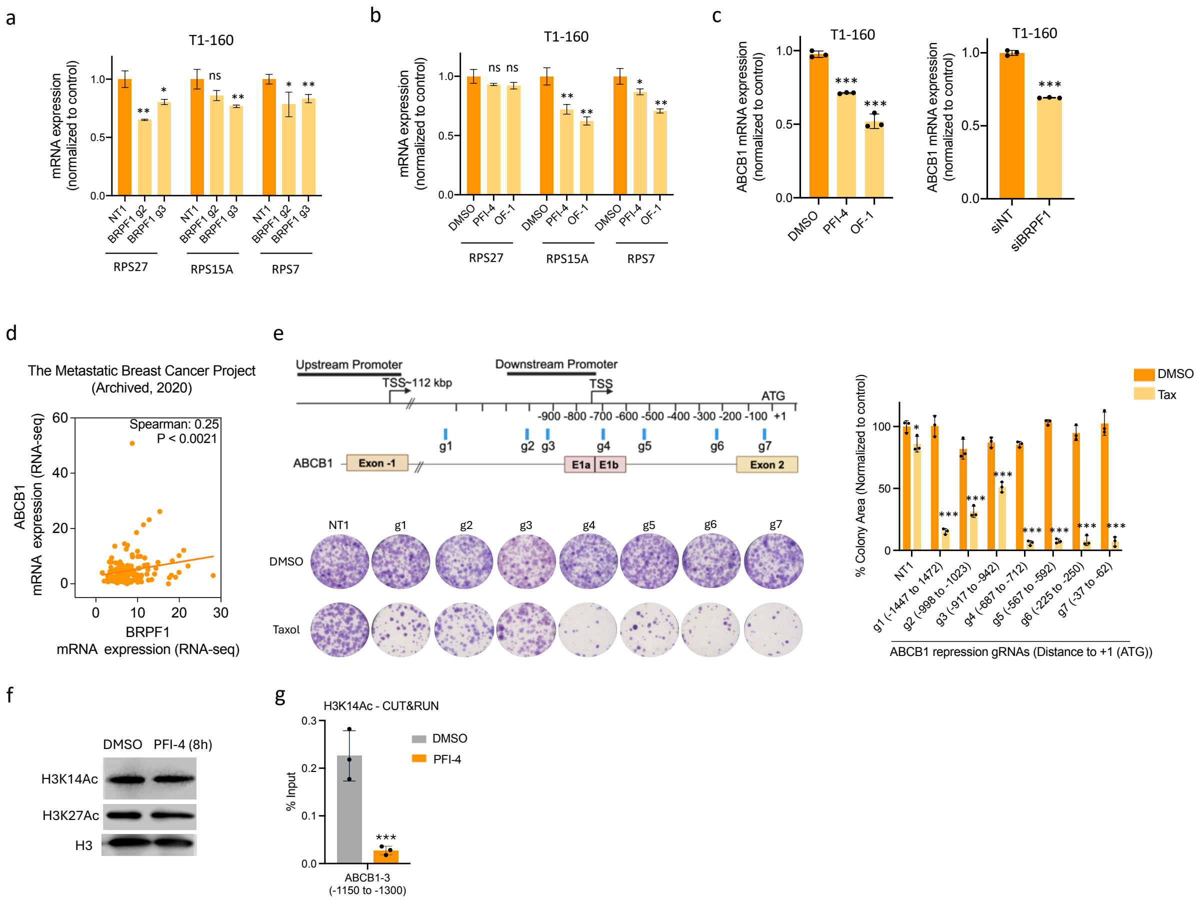

Supplementary Figure 8. mRNA expression of selected ribosome-related genes upon BRPF1 loss or inhibition. a. mRNA expression levels of ribosome-related genes in BRPF1 KO T1-160 cells. **b.** mRNA expression levels of ribosome-related genes in BRPF1 inhibitor treated T1-160 cells. **c**. ABCB1 mRNA levels upon 72 hours of PFI-4 and OF-1 or 48 hours of siBRPF1 treatment on T1-160 cells. **d.** Correlation analysis of BRPF1 and ABCB1 mRNA expression in metastatic breast cancer patients. mRNA expression levels were obtained from publicly available datasets in cBioPortal. The correlation was assessed using Spearman's rank correlation test, and the trend line represents the linear regression fit. **e**. Schematic representation shows sgRNAs designed for CRISPRi-mediated inhibition at the BRPF1 binding regions of the *ABCB1* promoter. Representative colony formation assay showing the impact of CRISPRi-mediated inhibition at different target regions on Taxol-resistant T1-160 cells in the absence and presence of 125 nM Taxol. Bar plot represents the quantification of colony area following CRISPRi experiment. **f.** Western blot analysis showing global H3K14Ac and H3K27Ac levels upon 8 hours of PFI-4 treatment on T1-160 cells. **g.** CUT&RUN-qPCR result showing significant decrease in H3K14Ac levels on ABCB1 promoter upon PFI-4 treatment. P values determined by two-tailed Student's t-test in comparison to control group; *p < 0.05, **p < 0.01, ***p < 0.001.

SUPPLEMENTARY TABLES

Supplementary Table 1: Antibodies used in this study

**Primary Antibody**	Brand, Cat no
Total PARP	Abcam, ab74290
Cleaved PARP	Cell Signaling, 9541
Cleaved Caspase 3	Cell Signaling, 9664
GAPDH	Abcam, ab9485
ABCB1	Cell Signaling, 13978
Tubulin	Abcam, ab15246
BRPF1	Novus, NBP2-15620
H3	Abcam, ab9049
H3K27Ac	Cell Signaling, 4353
H3K23Ac	Millipore, 07-355
H3K14Ac	Cell Signaling, 7627
H3K9Ac	Cell Signaling, 9649
Puromycin	Merck Millipore, MABE342
**Secondary Antibody**	**Brand, Cat no**
Goat anti-rabbit	Abcam, ab97051

Supplementary Table 2: qPCR primers used in this study

Gene	Forward Primer (5' to 3')	Reverse Primer (5' to 3')
GAPDH	AGCCACATCGCTCAGACAC	GCCCAATACGACCAAATCC
BRPF1	CCACACTGAAGATGCAGCCGA	CCGCAGTGCCGTCATCTCTT
BRPF2	CAGCGGAAGAAGCAGTTTGTG	TCTTTGGCAGCCTTCATCTCC
BRPF3	AGAACATCGGCTATGACCCC	CACCTCTGGGGACAAATGGG
RUNX1	GGTTTCGCAGCGTGGTAAAA	GCACTGTGGGTACGAAGGAA
RUNX2	CCGAGACCAACAGAGTCATTTA	ACATGGTGTCACTGTGCTGAA
JADE3	AGACACCGTTCCACAGCCTTCT	ATGTAGCCAGGCTCTGTGGTGT
MEAF6	GCGGCTCTTCAGTAAATCCTCG	GGAGAAGTGTCACTTTCCGTCC
ING5	CAGAACGCCTACAGCAAGTGCA	CAGGTCTGCATCAAGCCTTCGA
KAT6A	CTTCAGTGAGAGCAGCGAGGAG	GTGGTGTTTGCGCTTTCGGACT
KAT6B	GGCAAGGATTTGGACGGTTTCTC	CGCTCTTCCAATATGCCAGGTAG
KAT7	TCCATCTCAGGATGCCCACTGT	GTCATCTTGCCTGTGAGACAGC
ABCB1	ACAGAGGGGATGGTCAGTGT	TCACGGCCATAGCGAATGTT
ABCC1	CCGCTCTGGGACTGGAATG	ATGTAGCCTCGGTCATGTCG
ABCC3	TATGCCCCCGATGAGGACCA	GACAGGGCACTCAGCTGTCTCA

Supplementary Table 3: Composition of Chemical Probe Library and working concentrations of drugs in screens

No	Source Name	Class/Target	Working Concentration (µM)
1	AMI-1	Arginine methyltransferase - PRMT	50
2	SGC707	Arginine methyltransferase - PRMT3	1
3	TP-064	Arginine methyltransferase - PRMT4	1
4	TP-064N	Arginine methyltransferase - PRMT4	1
5	MS049	Arginine methyltransferase - PRMT4, PRMT6	1
6	MS409N	Arginine methyltransferase - PRMT4, PRMT6	1
7	GSK591	Arginine methyltransferase - PRMT5	1
8	LLY-283	Arginine methyltransferase - PRMT5	1
9	MS023	Arginine methyltransferase - Type I PRMTs	1
10	GSK8814	Bromodomains - ATAD2	10
11	GSK8815	Bromodomains - ATAD2	10
12	GSK2801	Bromodomains - BAZ2A, BAZ2B	1
13	BAZ2-ICR	Bromodomains - BAZ2A, BAZ2B	1
14	BAY-299	Bromodomains - BRD1, TAF1	1
15	RVX-208	Bromodomains - BRD2, BRD3, BRD4, BRDT (BET, BD2)	5
16	(+)-JQ1	Bromodomains - BRD2, BRD3, BRD4, BRDT (BET)	1
17	PFI-1	Bromodomains - BRD2, BRD3, BRD4, BRDT (BET)	5
18	I-BET	Bromodomains - BRD2/3/4	1
19	I-BRD9	Bromodomains - BRD9	10
20	TP-472	Bromodomains - BRD9	1
21	TP-472N	Bromodomains - BRD9	1
22	LP99	Bromodomains - BRD9, BRD7	1
23	BI-9564	Bromodomains - BRD9, BRD7	1
24	GSK6853	Bromodomains - BRPF1/2/3	1
25	GSK9311	Bromodomains - BRPF1/2/3	1
26	PFI-4	Bromodomains - BRPF1B	1
27	CBP/BRD4 (0383)	Bromodomains - CBP, BRD4(1)	5
28	NVS-CECR2-1	Bromodomains - CECR2	1
29	NVS-CECR2-C	Bromodomains - CECR2	1
30	I-CBP112	Bromodomains - CREBBP, EP300	1
31	SGC-CBP30	Bromodomains - CREBBP, EP300	1
32	(-)-JQ1 (inactive)	Bromodomains - Negative control	1
33	Bromosporine	Bromodomains - pan-Bromodomain	1
34	OF-1	Bromodomains - pan-BRPF	5
35	NI-57	Bromodomains - pan-BRPF	1
36	GSK4027	Bromodomains - PCAF, GCN5	1
37	GSK4028	Bromodomains - PCAF, GCN5	1
38	L-Moses	Bromodomains - PCAF, GCN5	1
39	D-Moses	Bromodomains - PCAF, GCN5	1
40	SMARCA	Bromodomains - SMARCA, PB1	2.5
41	PB1/SMARCA	Bromodomains - SMARCA, PB1	1

42	PFI-3	Bromodomains - SMARCA2/4, PB1(5)	1
43	TRIM24/BRPF	Bromodomains - TRIM24/BRPF	10
44	GSK864	Dehydrogenase	5
45	5-Azacitidine	DNA methyltransferase (DNMT)	10
46	5-Azadeoxycitidine	DNA methyltransferase (DNMT) - DNMT1/3	5
47	CXD101	HDAC	1
48	PCI-24781	HDAC	10
49	Romidepsin	HDAC	1
50	Mocetinostat	HDAC	10
51	CI-994	HDAC - 1,2,3,(8)	1
52	Valproic acid	HDAC - aliphatic acid compounds	1000
53	RGFP966	HDAC - HDAC3	10
54	Rocilinostat	HDAC - HDAC6	10
55	Tubastatin A HCl	HDAC - HDAC6	10
56	PCI-34051	HDAC - HDAC8	5
57	Belinostat	HDAC - hydroxamic acids	5
58	SAHA	HDAC - hydroxamic acids	2.5
59	Trichostatin A	HDAC - hydroxamic acids - Class I & II	0.5
60	Entinostat	HDAC - ortho-amino anilides	0.5
61	EX 527	HDAC - SIRT1	1
62	SRT1720	HDAC - SIRT1 (indirect) activator	1
63	AGK2	HDAC - SIRT2	10
64	TMP269	HDAC -4, 5, 7 &9	10
65	TMP195	HDAC -4,5,7,9	1
66	Santacruzamate	HDAC 2	50
67	C646	Histone acetyltransferase (HAT) p300/CBP	1
68	A-485	Histone acetyltransferase (HAT) p300/CBP	1
69	A-486	Histone acetyltransferase (HAT) p300/CBP	1
70	Methylstat (Ester)	Histone demethylase	2.5
71	KDOBA67	Histone demethylase	10
72	KDM5-C70	Histone demethylase - JARID1	10
73	ML324	Histone demethylase - JMJD2E	5
74	(E)-JIB-04	Histone demethylase - Pan JmjC	0.05
75	SGC0946	Histone methyltransferase - DOT1L	7.5
76	GSK343	Histone methyltransferase - EZH2	3
77	UNC1999	Histone methyltransferase - EZH2	1
78	UNC2400	Histone methyltransferase - EZH2	1
79	CPI-360	Histone methyltransferase - EZH2 and EZH1	10
80	CPI-169	Histone methyltransferase - EZH2, EZH1	10
81	UNC0642	Histone methyltransferase - G9a, GLP	1
82	UNC0638	Histone methyltransferase - G9a, GLP	1
83	A-366	Histone methyltransferase - G9a, GLP	2
84	PFI-2	Histone methyltransferase - SETD7	2
85	LLY-507	Histone methyltransferase - SMYD2	1
86	BAY-598	Histone methyltransferase - SMYD2	1
87	PFI-5	Histone methyltransferase - SMYD2	1
88	Chaetocin	Histone methyltransferase - SUV39H1	0.05

89	A-196	Histone methyltransferase - SUV420H1/H2	1
90	K00135	Kinase inhibitor - ATP competitive - PIM	1
91	5-Iodotubercidin	Kinase inhibitor - ATP mimetic - Haspin	1
92	SGI-1776	Kinase inhibitor - Haspin	10
93	CHR-6494	Kinase inhibitor - Haspin	1
94	KDOAM-25a	Lysine demethylases - JARID	1
95	KDOAM32	Lysine demethylases - JARID	1
96	GSK J4	Lysine demethylases - JMJD3, UTX, JARID1B	10
97	KDOPZ-32a	Lysine demethylases - KDM5	1
98	Tranylcypromine	Lysine demethylases - LSD1	20
99	GSK-LSD1	Lysine demethylases - LSD1 - irreversible	0.5
100	GSK2879552	Lysine demethylases - LSD1	10
101	GSK J5 (inactive)	Lysine demethylases - Negative control	10
102	IOX1	Lysine demethylases - pan-2-OG - (5-carboxy-8HQ)	40
103	KDOOA012000	Lysine demethylases KDM2	1
104	A-395	Methyl Lysine Binder - EED	1
105	A-395N	Methyl Lysine Binder - EED	1
106	UNC1215	Methyl Lysine Binder - L3MBTL3	5
107	OICR-9429	Methyl Lysine Binder - WDR5	1
108	TDO20824a	Methyl Lysine Binder/tudor domain - Spin1	1
109	TDO20826a	Methyl Lysine Binder/tudor domain - Spin1	1
110	GSK484	Peptidyl arginine deiminase (PAD4)	1
111	GSK106	Peptidyl arginine deiminase (PAD4)	1
112	Olaparib	Poly ADP ribose polymerase (PARP)	1
113	Rucaparib	Poly ADP ribose polymerase (PARP)	10
114	IOX2	Prolyl-Hydroxylases - PHD2 (EGLN1)	10
115	MAZ1805		1
116	MAZ1392		1
117	Bortezomib		0.1
118	Carfilzomib		0.1

Supplementary Table 4: Sequences of sgRNAs used in this study

Gene	Forward Sequence (5' to 3')	Reverse Sequence (5' to 3')
Non-targeting (NT1)	GACGGAGGCTAAGCGTCGCAA	TTGCGACGCTTAGCCTCCGTC
ABCB1_g1	TTGGACTGTCAGCTGCTGTC	GACAGCAGCTGACAGTCCAA
BRPF1_g1	AGGGTGACTGCAGGCAACGG	CCGTTGCCTGCAGTCACCCT
BRPF1_g2	CCAACCGCCTGACCATCCAA	TTGGATGGTCAGGCGGTTGG
BRPF1_g3	TGAGTACCTAATGGACCGAC	GTCGGTCCATTAGGTACTCA
BRPF2_g1	CGACTCACCGGCTGCGATCC	GGATCGCAGCCGGTGAGTCG
BRPF2_g2	GCAGCAGTCTCTGATCGACG	CGTCGATCAGAGACTGCTGC
BRPF3_g1	AGAACCAGTCAACTTGAGTG	CACTCAAGTTGACTGGTTCT
BRPF3_g2	GAGCGCCATGCGGTCCAGTG	CACTGGACCGCATGGCGCTC
KAT6A_g1	TTCACTCGAACCGTTAGTTC	GAACTAACGGTTCGAGTGAA
KAT6B_g1	TGAAAGACGGACCGCAGTAC	GTACTGCGGTCCGTCTTTCA
MEAF6_g1	ATGTATGGCAATATTATTCG	CGAATAATATTGCCATACAT
ING5_g1	CTTCCAGCTGATGCGAGAGC	GCTCTCGCATCAGCTGGAAG
JADE3_g1	TCAGCATTGCTTGTCCTGAG	CTCAGGACAAGCAATGCTGA
KAT7_g1	TCTCATCGTGAGATACATTG	CAATGTATCTCACGATGAGA
ARID1A_g1	AATACTCACAGGCAAGCTGG	CCAGCTTGCCTGTGAGTATT
ARID1A_g2	ATGGTCATCGGGTACCGCTG	CAGCGGTACCCGATGACCAT
GATAD1_g1	AGAGTAAGCAGGAAATTCAC	GTGAATTTCCTGCTTACTCT
GATAD1_g2	CGTGACTTGAAATACTCAGA	TCTGAGTATTTCAAGTCACG
SMARCE1_g1	ACCAACAGCCGGGTCACGGT	ACCGTGACCCGGCTGTTGGT
SMARCE1_g2	TATGTAAGCAAGGTACGCGG	CCGCGTACCTTGCTTACATA
KMT2A_g1	AGAAAGGACGTCGATCGAGG	CCTCGATCGACGTCCTTTCT
KMT2A_g2	TCAGAGTGCGAAGTCCCACA	TGTGGGACTTCGCACTCTGA
MEN1_g1	CATGCGCTGTGACCGCAAGA	TCTTGCGGTCACAGCGCATG
MEN1_g2	CCAGGCATGATCCTCAGACA	TGTCTGAGGATCATGCCTGG
ARNTL_g1	CTGGACATTGCGTTGCATGT	ACATGCAACGCAATGTCCAG
ARNTL_g2	TTAGAATATACAGAACACCA	TGGTGTTCTGTATATTCTAA
BRD8_g1	AGGAGGTGATTATCCACTTG	CAAGTGGATAATCACCTCCT
BRD8_g2	ATAAGTACCTATATCTCTCC	GGAGAGATATAGGTACTTAT
MLLT6_g1	AATCTCAGGAGCGAGCAGCC	GGCTGCTCGCTCCTGAGATT
MLLT6_g2	AGCTTGCTATGGCATCGTTC	GAACGATGCCATAGCAAGCT
BRD3_g1	CGACGTGACGTTTGCAGTGA	TCACTGCAAACGTCACGTCG
BRD3_g2	GAGGAGAGCTCTTCGGACTC	GAGTCCGAAGAGCTCTCCTC
PPP2CA_g1	AATAAAAGTCATACCTCATG	CATGAGGTATGACTTTTATT
PPP2CA_g2	GGTATATCTCCTCGAGGAGC	GCTCCTCGAGGAGATATACC
CHD8_g1	CTGTCTTCTACACTACCGTG	CACGGTAGTGTAGAAGACAG
CHD8_g2	CTTTAATCCAGACTACGTAG	CTACGTAGTCTGGATTAAAG

Supplementary Table 5: CUT&RUN-qPCR primers used in this study

Gene	Forward Primer (5' to 3')	Reverse Primer (5' to 3')
MYT1	CCTGCCGTGTGCTGTTTTT	CACAACATGTCCCCTGGAATC
ABCB1-1 (-650 to -800)	CTTGGAAGAGCCGCTACTCG	TCTGTGGTGAGGCTGATTGG
ABCB1-2 (-780 to -900)	CACCACAGATGACTGCTCCC	CGCGTTTCTCTACTTGCCCT
ABCB1-3 (-1150 to -1300)	TGCAGTGGTCTTTCTTCAGCA	ACACGGGCATTGATCTGACG
ABCB1-4 (-1500 to -1650)	TGCTAACTCACATCAGAGCTTTT	ACATCCTCAGACTATGCAGTAAA
ABCB1-4 (~112 kbp)	TTCTCTCTGTGACAGCTCAGT	AGCACAAATTGAAGGAAGGAGT
RPL24	AAGTCCATCAGGGAGGGTGT	CGCCATGAAGTAAGCACGAC

Supplementary Table 6: q-RT-PCR from genomic DNA primers used in this study

Gene	Forward Primer (5' to 3')	Reverse Primer (5' to 3')
GAPDH	CGGCTACTAGCGGTTTTACG	AAGAAGATGCGGCTGACTGT
ABCB1	AGATCTACCAGGACGAGTGAGAAAA	AACAGTCAGTTCCTATATCCTGTGTCT

Supplementary Table 7: Sequences of sgRNAs used for ABCB1 CRISPRi experiment

Gene	Forward Primer (5' to 3')	Reverse Primer (5' to 3')
ABCB1_repres_gRNA-1	GTTTGAAGTAAATAGTGGAC	GTCCACTATTTACTTCAAAC
ABCB1_repres_gRNA-2	TAACTACAGGACGTAGTTAA	TTAACTACGTCCTGTAGTTA
ABCB1_repres_gRNA-3	CGCGCATCAGCTGAATCATT	AATGATTCAGCTGATGCGCG
ABCB1_repres_gRNA-4	GCCGCTACTCGAATGAGCTC	GAGCTCATTCGAGTAGCGGC
ABCB1_repres_gRNA-5	CTACCTCGCGCTCCTTGGAA	TTCCAAGGAGCGCGAGGTAG
ABCB1_repres_gRNA-6	CGTCCGCGGCGACTGGAACC	GGTTCCAGTCGCCGCGGACG
ABCB1_repres_gRNA-7	TGCATGAGCCTCAGGCGCGC	GCGCGCCTGAGGCTCATGCA
